# The First Genomic and Proteomic Characterization of a Deep-Sea Sulfate Reducer: Insights into the Piezophilic Lifestyle of *Desulfovibrio piezophilus*

**DOI:** 10.1371/journal.pone.0055130

**Published:** 2013-01-30

**Authors:** Nathalie Pradel, Boyang Ji, Grégory Gimenez, Emmanuel Talla, Patricia Lenoble, Marc Garel, Christian Tamburini, Patrick Fourquet, Régine Lebrun, Philippe Bertin, Yann Denis, Matthieu Pophillat, Valérie Barbe, Bernard Ollivier, Alain Dolla

**Affiliations:** 1 Aix-Marseille Université, Université du Sud Toulon-Var, CNRS/INSU, IRD, MIO, UM110, Marseille, France; 2 Aix-Marseille Université, CNRS, LCB, UMR 7283, Marseille, France; 3 UMR 6236, Faculté de Médecine, Marseille, France; 4 Laboratoire de Finition C.E.A., Institut de Génomique – Genoscope, Evry, France; 5 Plate-forme Protéomique, CIML, Marseille, France; 6 Plate-formes Protéomique et Transcriptomique FR3479, IBiSA Marseille-Protéomique. IMM - CNRS, Marseille, France; 7 UMR 7156, CNRS, Université Louis Pasteur, Strasbourg, France; University of Florida, United States of America

## Abstract

*Desulfovibrio piezophilus* strain C1TLV30^T^ is a piezophilic anaerobe that was isolated from wood falls in the Mediterranean deep-sea. *D. piezophilus* represents a unique model for studying the adaptation of sulfate-reducing bacteria to hydrostatic pressure. Here, we report the 3.6 Mbp genome sequence of this piezophilic bacterium. An analysis of the genome revealed the presence of seven genomic islands as well as gene clusters that are most likely linked to life at a high hydrostatic pressure. Comparative genomics and differential proteomics identified the transport of solutes and amino acids as well as amino acid metabolism as major cellular processes for the adaptation of this bacterium to hydrostatic pressure. In addition, the proteome profiles showed that the abundance of key enzymes that are involved in sulfate reduction was dependent on hydrostatic pressure. A comparative analysis of orthologs from the non-piezophilic marine bacterium *D. salexigens* and *D. piezophilus* identified aspartic acid, glutamic acid, lysine, asparagine, serine and tyrosine as the amino acids preferentially replaced by arginine, histidine, alanine and threonine in the piezophilic strain. This work reveals the adaptation strategies developed by a sulfate reducer to a deep-sea lifestyle.

## Introduction

Sulfur cycling is an important biogeochemical process on Earth, especially in oceans where the majority of sulfur is present as sulfate (SO_4_^2−^). Several data clearly indicate that microbial mediated sulfate reduction is a major terminal electron accepting process in the degradation of organic matter in marine sediments [1], [2], [3], [4]. It thus emphasizes the importance of sulfate-reducing microorganisms in both the sulfur and carbon cycles. Because 60% of the Earth’s surface is composed of deep-sea environments, we can postulate that a significant part, and even the majority of sulfur cycling occurs in the deep water ecosystem and, therefore, deep-sea sulfate-reducing microorganisms should be considered as key actors in Earth’s sulfur cycling. Metagenomic studies have revealed that sulfate-reducing bacteria (SRB) are largely represented among the microorganisms that inhabit deep-sea environments [5], [6], [7]. However, a few thermophilic and mesophilic SRB have been isolated from deep-sea environments [8], [9], [10], [11], [12], [13]. Among them, the *Desulfovibrio* genus is well-represented with *D. profundus*, *D. hydrothermalis*, and *D. piezophilus* [11], [12], [13].

Among the physico-chemical parameters that are characteristic of deep-sea environments, one of the most important is the increase of the hydrostatic pressure with depth (1 MPa for every 100 m depth). Therefore, hydrostatic pressure could be an important factor that influences sulfate reduction and the global sulfur cycle, especially in the deep ocean. Within these ecosystems, the hydrostatic pressure can reach up to 110 MPa (Mariana Trench) with an average hydrostatic pressure of approximately 38 MPa, corresponding to depth of 3800 m. Organisms that experience optimal growth at hydrostatic pressure higher than atmospheric pressure (0.1 MPa) are called "piezophiles" [14]. Until now, factors involved in piezophilic lifestyle have mainly been analyzed in two facultative anaerobic heterotrophic genera, *Photobacterium* and *Shewanella* [15], [16], [17], [18], [19]. However, these two genera do not accurately represent the complexity and diversity of the deep-sea ecosystems; anaerobes and SRB, as discussed above, are also important members of these biotopes that have to be considered [1], [20], [21], [22].

We recently isolated the sulfate-reducing anaerobe *Desulfovibrio piezophilus* C1TLV30^?^ from Mediterranean deep-sea wood falls at a depth of 1700 m [13]. Because appreciation of the occurrence of deep-sea wood falls is increasing, it makes them important organic-rich biotopes in these depth environments [23]. The degradation of the wood falls is expected to be successive, beginning with cellulose and other wood components degradation by heterotrophic bacteria. Diverse microbial communities would then develop through fermentation of other compounds; sulfate-reducing bacteria would, in turn, use the produced H_2_ and led to an enrichment of hydrogen sulfide at the surface of the wood. The mesophilic piezophile *D. piezophilus*, which grows optimally at 30°C and 10 MPa, provides thus an unique opportunity to study the adaptation strategies of SRB to hydrostatic pressure.

In this paper, we report the genomic sequence of *D. piezophilus* CITLV30^T^ and a comparative analysis of its sequence with the genomes of non-piezophilic *Desulfovibrio* species. In addition, we performed a differential proteome analysis after growth at atmospheric (0.1 MPa) and high (10 MPa) hydrostatic pressure. This analysis highlighted the importance of several gene clusters and metabolic pathways, such as amino acid metabolism and transport, in the adaptation to hydrostatic pressure and provided new insight into the piezophilic lifestyle and the evolution of the genus *Desulfovibrio*.

## Results

### General Features of the *D. piezophilus* C1TLV30^T^ Genome

**Figure 1:**
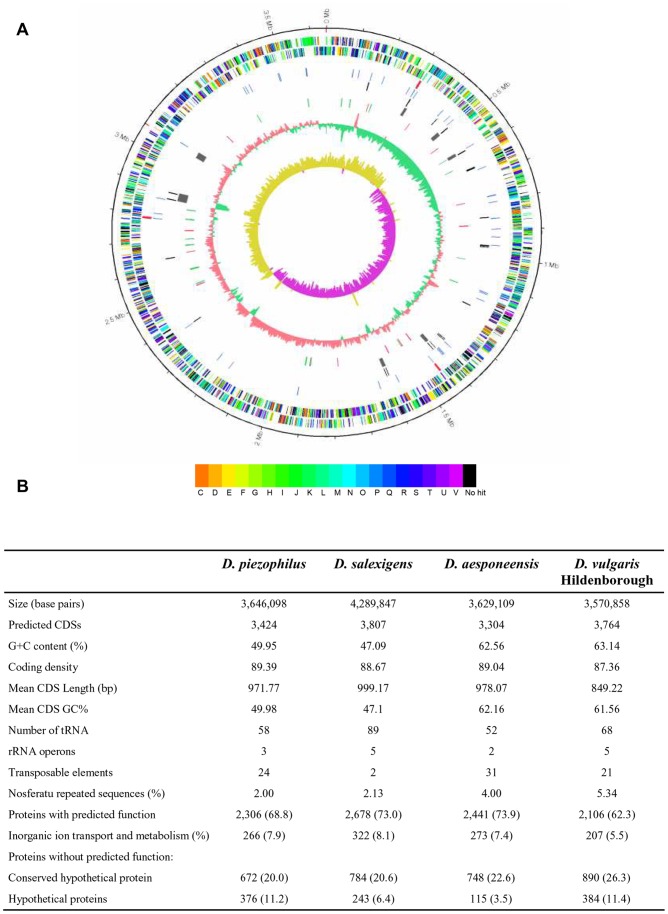
Overview of the *D. piezophilus* genome. **A.** Representation of the circular chromosome of the *D. piezophilus* strain C1TLV30^T^ in the form of concentric circles. The outer scale designates the coordinates in 500,000 base pairs, and the red ticks indicate physical gaps between two contigs in the same scaffold. Circles display (from outside to inside) in: 1) the predicted coding sequences on the plus strand; 2) the predicted coding sequences on the minus strand (the coding sequences are colored according to their putative roles as determined by COG classification);. 3) rRNA; 4) tRNA; 5) transposable elements; 6) predicted genomic islands (GI, Table S1); 7) proteins that vary in abundance depending on the pressure (red, proteins more abundant at 10 MPa; green, proteins less abundant at 10 MPa); 8) GC content using a 5 kbp sliding window with step size of 1 kbp (red indicates that the GC content is higher than the average GC content and green indicates that the GC content is lower than the average GC content); 9) GC skew (calculated with the same parameters used for the GC content), with red indicating levels greater than 0 and green indicating levels less than 0. **B.** General features of the *D. piezophilus* C1TLV30^T^ genome compared with those of three other *Desulfovibrio* sp. *D. aespoeensis* and *D. salexigens* were chosen as they belong to the same phylogenetic group (Fig. S3) and *D. salexigens* is also a mesophilic marine strain as *D. piezophilus*. *D. vulgaris* Hildenborough was introduced as model organism for *Desulfovibrio* species [47]. Twenty-one amino acid residues are represented by the tRNA genes in each organism. The numbers in parentheses are the percentages of the total number of proteins.

The *D. piezophilus* C1TLV30^T^ genome consists of a single circular molecule of 3,644,098 bp with a GC content of approximately 50% (Fig. 1). Although the GC bases are not evenly distributed throughout the chromosome, the average GC content of *D. piezophilus* is quite low compared to the values found in other *Desulfovibrio* species, with the exception of *D. salexigens*. Seven genomic islands that range from 8 to 30 kbp were identified in the C1TLV30^T^ genome (Fig. 1; Table S1). This number is in the same range as for other *Desulfovibrio* species (Table S8). The genes in these islands encode mainly prophage-like elements, proteins of unknown function, and hypothetical proteins. Very few insertion sequences (IS)/transposase elements were found (Fig. 1B), contrary to *Photobacterium profundum* SS9 that has many [24]. It indicates that *D. piezophilus* genome has not been subjected to many genomic rearrangements during its evolution. The low number of IS in *D. piezophilus* could be one characteristic of this phylogenetic group, as it is in the same range as in other *Desulfovibrio* species (Fig. 1B).

The *D. piezophilus* genome contains 3,424 coding DNA sequences (CDSs). Approximately 77.16% of the CDSs are classified in at least one Cluster of Orthologous Group of Proteins (COGs). Compared to representative non-piezophilic *Desulfovibrio* genomes (*D. salexigens, D. vulgaris,* and *D. aespoeensis*), *D. piezophilus* has a larger proportion of genes that encode proteins involved in energy production and conversion (C), cell cycle control, cell division, chromosome partitioning (D), amino acid transport and metabolism (E), and post-translational modification, protein turnover, chaperones (O) inorganic ion transport and metabolism (P), secondary metabolites biosynthesis, transport and catabolism (Q) (Fig. S1). Those functional classes may be relevant for the cellular adaptation of *D. piezophilus* to hydrostatic pressure. Conversely, proteins that are involved in coenzyme transport and metabolism (H) are less represented in *D. piezophilus* than in other *Desulfovibrio* spp.

### Energy Metabolism and Sulfate Respiration in *D. piezophilus*

The main mode of energy generation in *D. piezophilus* is sulfate reduction. As expected, the *D. piezophilus* genome contains genes that encode proteins that are required for sulfate reduction, including sulfate transport, activation to adenosine-5′-phosphosulfate (APS) and APS reduction (Table S2). Energy conservation in SRB involves a number of periplasmic and cytoplasmic enzymes, as well as transmembrane complexes [25], [26] that are detected in the *D. piezophilus* genome (Table S2). *Desulfovibrio* species are characterized by a very high level of c-type cytochromes [26]. Although the *D. piezophilus* genome does not contain an extensive number of c-type cytochrome-encoding genes, it encodes several multiheme c-type cytochromes and one monoheme cytochrome (Table S2). One of these cytochromes (DESPIv2_11010) is closely related to a cytochrome from the piezophile *Photobacterium profundum.* Notably, this cytochrome was expressed at significantly higher levels under high (10 MPa) than low (0.1 MPa) hydrostatic pressure conditions (Table S3).

### Lipid Biosynthesis Pathways in *D. piezophilus*

A change in membrane fluidity is a well-established response to hydrostatic pressure exposure. Unsaturated fatty acids have been shown to be important for the adaptation to hydrostatic pressure [19]. We have previously shown increases in the chain length of membrane fatty acids (FAs) and in the abundance of monounsaturated FAs when *D. piezophilus* was cultured at a high hydrostatic pressure [13]. All of the genes necessary for the biosynthesis of FAs, phospholipids and peptidoglycans are present in the *D. piezophilus* genome. Notably, the *fabF* gene (DESPIv2_11609), which encodes a β-ketoacyl-ACP synthase II involved in the production of monounsaturated FAs, is present in the *D. piezophilus* genome. Its activity has been suggested to be responsive to pressure in piezophilic bacteria [27]. More interestingly, the *ole1* gene (DESPIv2_10162), which encodes a stearoyl-CoA desaturase, is also present. The activity of *ole1* could increase the proportion of unsaturated FAs. It has been suggested that the *ole1* gene product was involved in membrane fluidity at high hydrostatic pressure [28]. In addition, it has been shown that the *P. profundum* SS9 *ole1* ortholog increased its expression when pressure rose from atmospheric to high hydrostatic pressure [29]. Because the other genes that were identified as likely to be involved in lipid biosynthesis are also found in non-piezophilic bacteria, changes in the membrane lipid composition of *D. piezophilus* could also be attributed to the regulation of the classical lipid biosynthesis pathways.

### Comparative Genomics Reveal Specific Gene Content and Functions in *D. piezophilus*

A comparative genomic approach was carried out by searching for homologs within the non-redundant (nr) database. Among the 3,424 CDSs, 241 ORFans specific to *D. piezophilus* were found (no hits *versus* nr), and additional 106 genes that were not related to genes found in other sulfate-reducing microorganisms were identified (Table S4). Among the *D. piezophilus*-specific genes, 33 are found in predicted genomic islands, particularly GI5 and GI6, which contain 17 and 7 genes, respectively. The 241 ORFans encode proteins of unknown function that could play important roles in the piezophilic capacity of *D. piezophilus*. Among the latter 106 genes, 12% encoded proteins belonging to either the P or E COG categories (metabolite or ion metabolism and transport), 11% to the K or L COG categories (transcription, recombination and repair mechanisms), and 44% encoded proteins that did not belong to any COG category. Among these genes, some could be related to the hydrostatic pressure adaptation in this strain. Regarding the organization of the 347 genes, 19 gene clusters (at least 3 genes) were identified (Table S4). Interestingly, phylogenetic analyses revealed that three clusters (DESPIv2_11206-11237, DESPIv2_11831-11838, and DESPIv2_12041-12113) included genes that are closely related to genes from microorganisms able to growth under high hydrostatic pressure, such as *Colwellia psychrerythraea* [30] or *Photobacterium profundum* [17] (Fig. S2 and Table S4). We then used qRT-PCR to determine whether the expression levels of these genes were dependent on hydrostatic pressure. The gene DESPIv2_11220, a representative of the gene cluster DESPIv2_11206-11237, appeared to be up-regulated when the cells were cultured under high (10 MPa) conditions compared with low (0.1 MPa, atmospheric pressure) hydrostatic pressure conditions, while the expression of the representative genes of the two other gene clusters, DESPIv2_12106 and DESPIv2_11838, were not affected by the pressure (Table S3).

To gain further insight into the piezophilic lifestyle in bacteria, we compared the distribution of the COG classes of *D. piezophilus* and *P. profundum*, the most well-studied piezophilic bacterium, to that of *D. salexigens*, the only non-piezophilic marine *Desulfovibrio* for which a genome sequence is available, and *D. vulgaris* Hildenborough, the best known *Desulfovibrio* strain. This comparison revealed 53 COGs that were present in both piezophilic strains but absent in the non-piezophilic strains (Table S5). Among them, the most highly represented functional categories are amino acid transport and metabolism (E), secondary metabolite biosynthesis, transport and catabolism (Q), general functional prediction only (R) and unknown function (S) categories. Interestingly, we found proteins involved in the tripartite TRAP-type mannitol/chloro compound transport system (COG4663, COG4664, and COG4665). TRAP transporters have been reported to be involved in several mechanisms used by bacteria to adapt to different habitats [31]. This suggests that the transport of solutes plays an important role in the piezophilic lifestyle of *D. piezophilus*.

### *D. piezophilus* has a Different Amino Acid Composition than other *Desulfovibrio* spp

**Figure 2:**
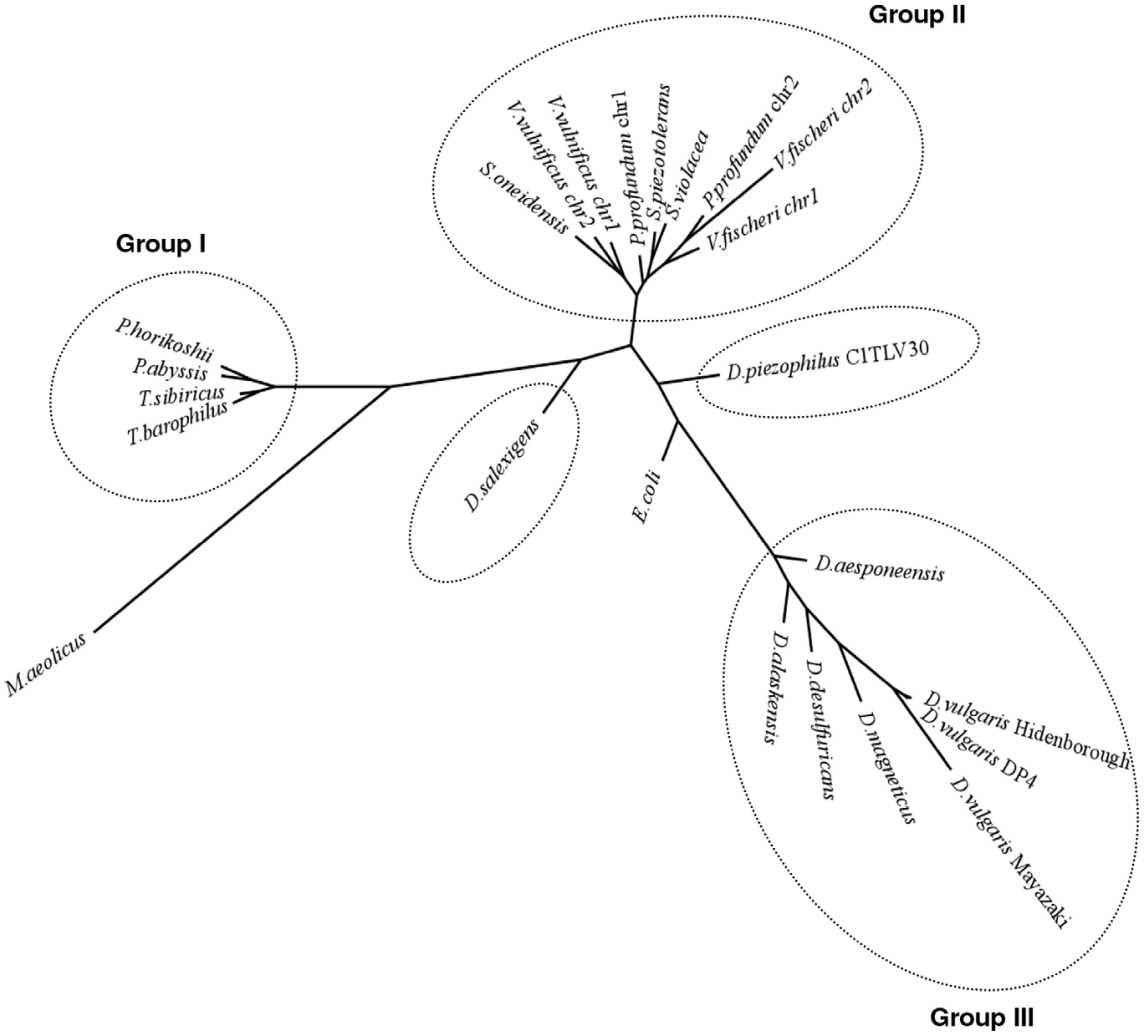
Clustering of piezophilic and non-piezophilic prokaryotes based on amino-acid composition. The neighbor-joining tree inferred from the amino acid composition of the selected genomes, including piezophilic prokaryotes. Based on the amino acid frequency, the distance matrix was calculated with the Pearson correlation distance, and the inferred tree was constructed with the Phylip package using the neighbor-joining (NJ) method. The list of organisms with their taxonomic affiliations and their GC and GC3 content is shown in Table S6. The organisms with similar amino acid compositions are circled with a dotted line.

A tree-based amino acid (AA) composition analysis of 21 selected strains, including piezophilic and non-piezophilic archaea, Gammaproteobacteria and *Desulfovibrio* organisms, revealed clusters of three main groups (Fig. 2). These microorganisms were chosen according to their taxonomic affiliation and their habitat (Fig. S3, Table S6). The first group (Group I) was composed of archaeal organisms and included known piezophiles (*Pyrococcus abyssi* and *Thermococcus barophilus*), while the second group (Group II) was composed of all of the analyzed Gammaproteobacteria (with three known piezophilic strains : *Photobacterium profundum*, *Shewanella piezotolerans*, *Shewanella violacea*). The third group (Group III) was composed of 7 *Desulfovibrio* strains. Interestingly, *D. piezophilus* and *D. salexigens* appeared in isolated branches at the depth level of *Desulfovibrio*. While known piezophilic bacteria and archaea shared similar AA compositions with their closely related phylogenetic strains, *D. piezophilus* exhibited an amino acid composition that was distinct from that of the other *Desulfovibrio* species. It is known that the GC content and the GC3 influence the AA composition of an organism [32], [33]. Because *D. piezophilus*, *D. salexigens,* some archaea (*e.g., P. abyssi*) and Gammaproteobacteria (*e.g., Vibrio vulnificus*) had similar average GC contents and GC3 (Table S6), these two factors could not be responsible for the observed clustering pattern. Taken together, these results strongly suggest that *D. piezophilus* has a different AA composition than other *Desulfovibrio* or piezophilic strains, while other known piezophilic strains have AA compositions similar to those of their most closely related non-piezophilic relatives (Fig. 2). This suggests that the AA composition of *D. piezophilus* is a specific adaptation for a piezophilic lifestyle.

To study the link between AA composition and piezophilic capabilities, we studied the site-specific AA replacements in orthologous proteins from *D. piezophilus* and *D. salexigens*. Because previous investigations have shown the importance of the amino acid composition for adaptations to temperature and salinity [34], [35], [36], we chose to compare these two *Desulfovibrio* strains (1,911 orthologous proteins containing a total of 632,496 AAs), as they are both mesophilic and marine strains (Table S6). We thus expect to avoid any bias that could had been introduced with respect to different optimal salinity and temperature of growth. Table 1 shows the distribution of the total AA substitutions regarding a single AA between the two compared organisms. The shaded boxes indicate the statistically significant AA substitutions (*P*<0.0005). These data revealed that aspartic acid (D), glutamic acid (E), lysine (K), asparagine (N), serine (S) and tyrosine (Y) were preferentially replaced (2.33%, 6.82%, 13.10%, 8.10%, 5.30% and 1.57%, respectively) by other AAs in *D. piezophilus* orthologs. In particular, E, K and N were substituted at the statistically highest binary exchange rate. All of these AA were replaced mainly by alanine (A), histidine (H), arginine (R) and threonine (T) in *D. piezophilus*. These replacements were less frequent when *D. salexigens* was compared with *D. vulgaris* Hildenborough, indicating that this analysis excludes bias due to random drift mutations.

**Table 1:** Number of AA substitutions for each AA in the comparison of 1,911 orthologous proteins between *D. piezophilus* and *D. salexigens.*

D. piezophilus																					
	AA	A	C	D	E	F	G	H	I	K	L	M	N	P	Q	R	S	T	V	W	Y
D. salexigens	A	20257	374	609	1124	212	1565	182	567	734	696	314	317	690	501	493	2167	1283	1389	52	103
	C	417	3674	45	40	84	95	30	89	42	139	68	59	33	30	82	260	175	212	14	44
	D	**708**	33	13857	2843	58	755	**227**	89	624	119	72	1054	**345**	446	**296**	759	**554**	140	9	42
	E	**1543**	51	**3217**	16624	66	**713**	**296**	213	1362	**331**	160	563	**529**	**1244**	**738**	**983**	**896**	**416**	21	58
	F	217	64	36	67	11404	86	186	487	52	**1400**	289	63	65	76	76	169	170	372	151	1264
	G	1509	121	741	612	62	24896	**144**	105	425	164	75	498	221	233	333	897	352	173	19	40
	H	170	26	142	209	155	88	4823	69	189	145	55	240	79	288	282	191	154	87	28	307
	I	527	97	72	169	494	89	67	14289	166	**3471**	774	79	113	126	169	172	523	4167	47	130
	K	**989**	57	**725**	**1452**	**96**	**556**	**353**	229	12658	**426**	233	713	**392**	**1253**	**2689**	**877**	**1001**	**401**	28	84
	L	641	164	120	280	1225	161	174	2854	286	27777	1943	103	219	329	356	258	505	2010	**142**	297
	M	293	56	79	141	275	72	67	740	191	2146	6418	79	57	185	152	145	243	547	46	89
	N	**491**	54	1090	629	63	**596**	**366**	108	685	**158**	80	7465	**161**	**419**	**429**	**881**	**581**	133	13	107
	P	717	32	264	406	55	212	89	115	283	254	56	109	13594	195	165	499	298	201	23	32
	Q	436	29	419	1057	59	221	308	108	805	358	167	274	177	6142	668	400	367	180	25	58
	R	437	67	257	495	73	293	333	137	1969	304	132	261	164	617	13408	433	373	172	65	89
	S	**2729**	234	813	807	114	**1033**	**260**	197	703	318	157	743	515	450	**534**	11048	**1915**	369	29	90
	T	1138	153	379	571	128	290	144	461	530	469	255	397	294	318	376	1630	11069	810	29	63
	V	1541	225	115	320	364	187	82	4079	271	2081	638	107	209	182	211	365	946	17069	39	122
	W	35	12	8	18	122	22	34	39	18	95	31	9	12	17	42	24	26	34	3310	117
	Y	**140**	50	65	81	1381	46	**371**	132	71	311	97	98	50	83	94	139	100	129	**169**	7621

Other studies have also reported that arginine and alanine were the “most piezophilic AAs” and that they played a major role in adaptation to the piezophilic lifestyle in archaea [37], [38]. Other specific AA substitutions (F *versus* L, G *versus* H, I *versus* L, and L *versus* W) were also found to be significant and thus could also be factors in the adaptation of *Desulfovibrio* sp. to hydrostatic pressure.?The observed substitutions did not induce any significant variation of the average isoelectric point value of the *D. piezophilus* CDSs (approximately 5), which was similar to the value calculated for other *Desulfovibrio* species, suggesting that this parameter is not involved in the adaptation of *D. piezophilus* to hydrostatic pressure.

### Effect of Hydrostatic Pressure on the Proteome of *D. piezophilus*

**Figure 3:**
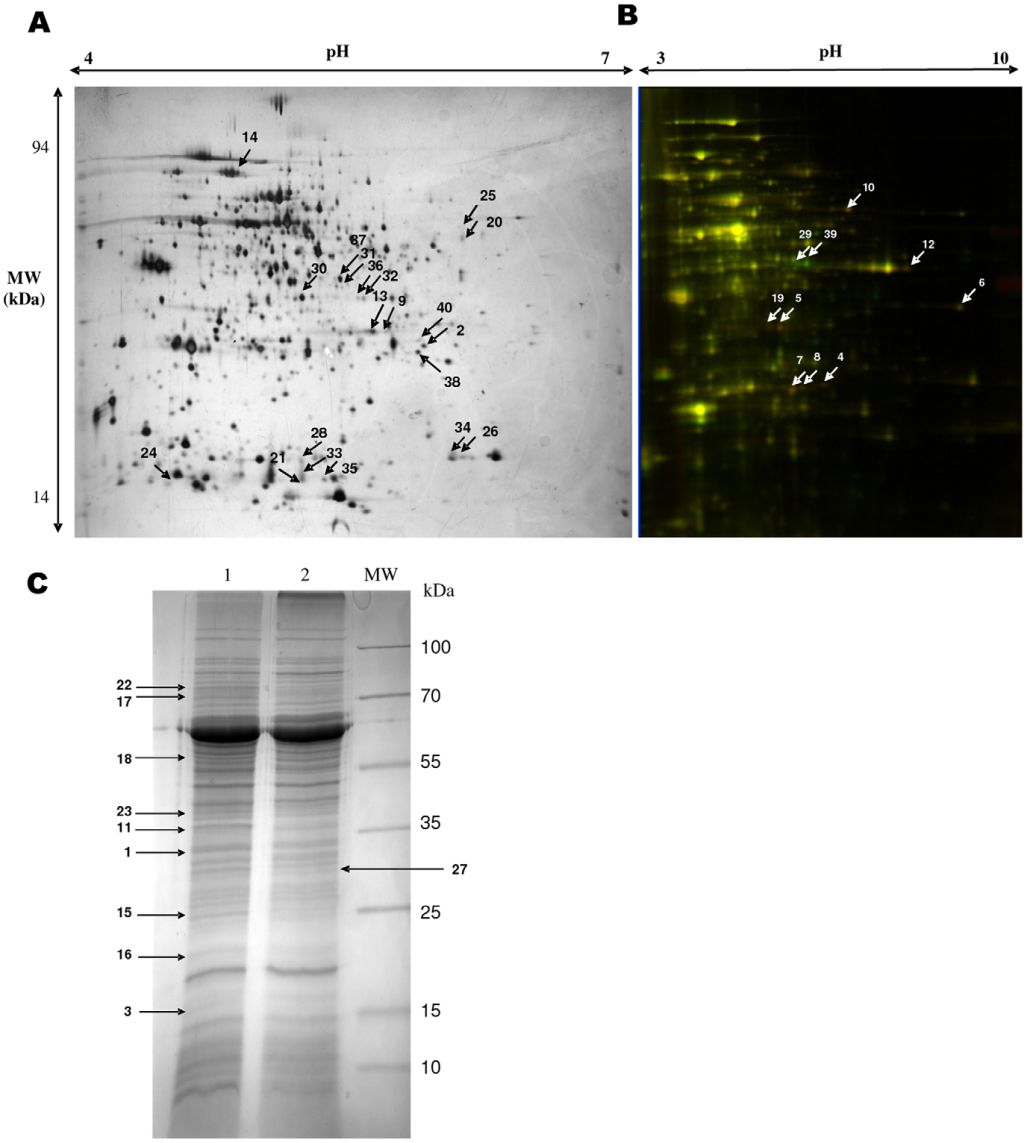
Differential proteome analyses depending on hydrostatic pressure. Proteome profiles of *D. piezophilus*. **A.** Overview of the proteome profile obtained by 2DE-gels of soluble proteins recovered from *D. piezophilus* grown at 10 MPa. The proteins (100 µg) were separated by 2D gel electrophoresis on a pH gradient of 4–7 and a 10–18% polyacrylamide denaturing gradient, and the gel was silver-stained. A total of 727 protein spots were detected. **B.** Overlapping DiGE images of the *D. piezophilus* proteome. The proteins were separated on a pH gradient of 3–10 and a 12% polyacrylamide denaturing gel (Cy3-green  = 10 MPa; Cy5-red  = 0.1 Mpa). A total of 940 protein spots were detected. **C.** Sodium dodecyl sulfate-polyacrylamide gel electrophoresis of the membrane protein fraction of *D. piezophilus* recovered at low (0.1 MPa, Lane 1) or high (10 MPa, Lane 2) hydrostatic pressure. Molecular weight standard, MW. The arrows indicate the positions of the differentially abundant proteins according to the hydrostatic pressure growth conditions.

Pressure-dependent variations in the proteome of *D. piezophilus* were evaluated by 1DE- and 2DE-gels for the membrane and soluble fractions, respectively (Fig. 3). The abundance of 24 protein spots or bands increased, while that of 16 protein spots or bands decreased, as a specific consequence of cell growth at low (0.1 MPa) compared to high (10 MPa) hydrostatic pressure. The corresponding identifications are indicated in Tables 2 and 3. As shown in Fig. 1A, the genes that encoded these proteins were scattered across the genome, demonstrating that there was no specific hotspot for high or low hydrostatic pressure genes within the *D. piezophilus* chromosome. Most of these proteins (11 proteins) were involved in AA transport and metabolism. Among them, the ABC transporter glutamine-binding protein GlnH (DESPIv2_10610) was more abundant under low hydrostatic pressure (Table 2). The gene encoding this protein was found to be up-regulated by qRT-PCR when cells were cultured under low hydrostatic pressure (Table S3). Interestingly, two copies of the gene that encodes GlnH (DESPIv2_10600 and DESPIv2_10610) have been found in the genome of *D. piezophilus*. A similar change in gene expression was also observed for DESPIv2_10600. The Gln transport thus could be an important mechanism for adaptation of *D. piezophilus* to hydrostatic pressure. It is also noteworthy that the abundance of HemK (DESPIv2_10174), a protein involved in glutamine transformation, was increased after growth at high hydrostatic pressure (Table 3). This observation emphasizes the possible importance of glutamate/glutamine metabolism and/or transport in the adaptation of the bacterium to hydrostatic pressure.

**Table 2:** *D. piezophilus* proteins with increased abundance under low (0.1 MPa) relative to high (10 MPa) hydrostatic pressure.

Spot number	Gene Accession	Annotation	COGs category	DA* (±0.2)
1	DESPIv2_11412#	Periplasmic [NiFe] hydrogenase small subunit, HynA-1	C	2.6
2	DESPIv2_11809	Adenylylsulfate reductase, α subunit, AprA	C	2.4
3	DESPIv2_12525#	ATP synthase subunit b, AtpF	C	2.2
4	DESPIv2_20239	Nitroreductase	C	1.5
5	DESPIv2_20445	Cobyrinic acid ac-diamide synthase	C	2.8
6	DESPIv2_20246	Extracellular solute-binding protein family 1, PotD	E	2.7
7	DESPIv2_10610	ABC transporter glutamine-binding protein GlnH	E	2.9
8	DESPIv2_10611	ATP-binding component of ABC superfamily, AapP	E	1.6
9	DESPIv2_10063	Polar amino acid ABC transporter subunit	E	4.5
10	DESPIv2_10036	Extracellular solute-binding protein, family 5, DppA	E	1.9
11	DESPIv2_12641#	Extracellular ligand-binding receptor	E	1.9
12	DESPIv2_10448	Triosephosphate isomerase, TpiA	G	2.4
13	DESPIv2_11436	Uncharacterized aldolase aq_1554	G	4.3
14	DESPIv2_12350	Ribonuclease, Rne/Rng family	J	4.4
15	DESPIv2_10222#	Peptidoglycan-associated lipoprotein Pal	M	2.0
16	DESPIv2_12413#	Outer membrane chaperone Skp (OmpH)	M	6.5
17	DESPIv2_12605#	Putative AsmA family protein	M	3.4
18	DESPIv2_11075#	Serine protease do-like DegP	O	9.6
19	DESPIv2_10232	Basic membrane lipoprotein	R	1.8
20	DESPIv2_12411	Tetratricopeptide domain protein	R	_
21	DESPIv2_10558	Conserved protein of unknown function	S	3.6
22	DESPIv2_11823#	Methyl-accepting chemotaxis sensory transducer	T	2.2
23	DESPIv2_10098#	Outer membrane efflux protein	U	2.0
24	DESPIv2_10982	Conserved protein of unknown function		3.7

* Differential abundance.

# Identified in the membrane fraction by 1DE-gels.

**Table 3:** *D. piezophilus* proteins with decreased abundance under low (0.1 MPa) relative to high (10 MPa) hydrostatic pressure.

Spot number	Gene Accession	Annotation	COGs category	DA* (±0.2)
25	DESPIv2_11808	Quinone-interacting membrane bound oxidoreductase, Flavin protein QmoA	C	3.9
26	DESPIv2_10343	Amino acid-binding ACT domain protein	E	2.4
27	DESPIv2_10492#	Extracellular solute-binding protein family 3	E	2.8
28	DESPIv2_11485	Extracellular solute-binding protein family 3	E	6.4
29	DESPIv2_12435	Alanine dehydrogenase	E	2.6
30	DESPIv2_12562	ATP phosphoribosyltransferase HisG	E	5.6
31	DESPIv2_10845	Ornithine carbamoyltransferase ArgF	F	1.8
32	DESPIv2_11386	TRAP dicarboxylate transporter DctP subunit	G	2.3
33	DESPIv2_10174	Modification methylase, HemK family	J	2.8
34	DESPIv2_11695	50 S ribosomal protein L19	J	3.8
35	DESPIv2_12480	Heat shock protein Hsp20	O	1.7
36	DESPIv2_20109	Conserved exported protein of unknown function	P	3.7
37	DESPIv2_11432	Putative Methyltransferase type 12	R	2.3
38	DESPIv2_12341	CBS domain-containing membrane protein	R	2.5
39	DESPIv2_10688	Sensor protein	T	2.6
40	DESPIv2_10745	Conserved protein of unknown function		2.4

* Differential abundance.

# Identified in the membrane fraction by 1DE-gels.

Three proteins involved in AA biosynthesis were found to be more abundant under high than low hydrostatic pressure: (*i*) alanine dehydrogenase, involved in alanine biosynthesis, (*ii*) the ATP phosphoribosyltransferase HisG (DESPIv2_12562), involved in histidine biosynthesis, and (*iii*) the ornithine carbamoyltransferase ArgF (DESPIv2_10845), involved in arginine biosynthesis. Therefore, as suggested by the genome analysis discussed above, AA transport and metabolism appear to play major roles in the adaptation of *D. piezophilus* to hydrostatic pressure.

The levels of three proteins that are involved in the sulfate reduction pathways and energy metabolism were found to vary in a pressure-dependent manner: the flavin protein QmoA (DESPIv2_11808), the [NiFe] hydrogenase small subunit HynA-1 (DESPIv2_11412), and the adenylylsulfate reductase alpha subunit AprA (DESPIv2_11809). Notably, none of the corresponding genes exhibited significant changes in expression (Table S3), suggesting that either translational regulation or protein stability changes in response to hydrostatic pressure.

The outer membrane chaperone OmpH (DESPIv2_12413) showed a large abundance increase (differential abundance (DA)  = 6.53) under low hydrostatic pressure. Similarly, the putative serine protease do-like DegP (DESPIv2_11075) was also found to be more abundant under this condition (DA  = 9.58). This protein was previously reported to be involved in the adaptation to atypical conditions [39], [40]. Thus, it could act as a chaperone in the adaptation to low hydrostatic pressure. Increases in chaperone proteins in response to low hydrostatic pressure has been previously shown in the piezophilic bacterium *P. profundum* SS9 [29]. The genes that encode OmpH and DegP did not exhibit significant pressure-dependent variations in expression levels (Table S3). The variations in the levels of these proteins could thus be linked to either the increased stability of the protein under low hydrostatic pressure or a translational regulatory mechanism.

DNA replication and translation processes have also been reported to play a role in the adaptation of microorganisms to hydrostatic pressure [18], [41], [42]. In the case of *D. piezophilus*, our differential proteomic analysis revealed few proteins involved in these processes, with the exception of a ribonuclease (DESPIv2_12350) involved in tRNA maturation and a 50S ribosomal protein L19 (DESPIv2_11695) that exhibited a significant variation in abundance that was dependent on the pressure. These variations could be linked to a translational regulation process that depends on hydrostatic pressure.

Five "unknown function proteins" could be good candidates for a specific adaptation to hydrostatic pressure. Among them, the gene encoding DESPIv2_20109 was up-regulated by a factor of 9.2 in high compared to low hydrostatic pressure conditions. Interestingly, DESPIv2_10558 was within GI3 and was located upstream of a gene-specific cluster (DESPIv2_10562 to 10565).

## Discussion

We present here the first sequenced genome of a piezophilic sulfate-reducing bacterium, *Desulfovibrio piezophilus* C1TLV30^T^. This genome sequence provides an important opportunity to better understand the influence of deep-sea marine microorganisms on the global sulfur cycle and to gain insights into the adaptation strategies utilized by these microorganisms to survive at different hydrostatic pressures.

Although the genomic characteristics of *D. piezophilus* are globally similar to those of other *Desulfovibrio* species, several differences may define or influence the piezophilic lifestyle of sulfate-reducing bacteria. Among the unique characteristics of the *D. piezophilus* genome are seven genomic islands that mainly include genes encoding proteins of unknown function. These genomic islands could be linked to the ability of the organism to adapt to its environment, and they may contain genes that are involved in piezophily. Additionally, the cluster regions DESPIv2_11206-11237, DESPIv2_11831-11838, and DESPIv2_12041-12113 include specific genes to *D. piezophilus* and genes closely related to genes found in the piezophilic *Photobacterium profundum* [29] and *Colwellia psychrerythraea,* this latter being able to growth under deep-sea pressure [30]. These genes are also expected to play important roles in the piezophilic lifestyle of *D. piezophilus*. Further studies, including genetic studies and analyses of protein structure/function relationships, will be required to determine the roles of these proteins in hydrostatic pressure adaptation.

In archaea, the piezophilic lifestyle has been directly associated with codon usage and tetranucleotide frequency [43]. However, this is not the case in *D. piezophilus*, in which these genomic patterns are similar to those of closely related non-piezophilic *Desulfovibrio* strains. However, we showed that the AA composition of *D. piezophilus* proteins is distinct from that of other *Desulfovibrio* species, suggesting that AAs are key factors in the adaptation to hydrostatic pressure. In addition, a comparative analysis with *D. salexigens* showed that AAs are replaced by A, R, H and T with significant frequency in the *D. piezophilus* orthologs. "Piezophilic AAs" like arginine have also been identified in the archaean *Pyrococcus* species [38], [43]. These AA replacements could be important for maintaining protein stability and/or activity at high hydrostatic pressure.

Our work identified specific genes/proteins that may be important for the adaptation of sulfate reducers to hydrostatic pressure. Enzymes involved in sulfate respiration (QmoA, HynA-1, and AprA), key components of the anaerobic energy metabolism of these microorganisms, are differentially abundant depending on the hydrostatic pressure. This suggests that the hydrostatic pressure has an impact on the dissimilatory sulfate reduction pathway in *D. piezophilus*. *S. violacea* adapts its respiratory components in response to alterations in pressure to conduct aerobic respiration [16]. One can conclude that the modulation of respiration pathways is a general mechanism by which microorganisms adapt to variations in hydrostatic pressure. The transport of AAs and various solutes and AA metabolism were also identified as major biological processes affected by hydrostatic pressure. Previous studies on piezophilic *Photobacterium* spp. and *Shewanella* spp. have also suggested that the adaptation to hydrostatic pressure involved changes in the transport of solutes [15], [19], [42]. These data suggest that the modification of proteins that are involved in transport represents a major general evolutionary adaptation strategy utilized by microorganisms in the deep-sea environment. Notably, the enzymes involved in the biosynthesis of arginine, histidine, and alanine were found to be more abundant under high than low hydrostatic pressure in *D. piezophilus*. These AAs may function to maintain the pH of the cytosol, as previously suggested by Abe [15]. In *P. profundum*, it has been suggested that "piezolites" could compensate for high hydrostatic pressure by maintaining osmotic pressure in the cell [44]. As AA transport and metabolism appear to be important cellular processes for the piezophilic lifestyle, we can propose that amino acids could also act as "piezolites" in *D. piezophilus*. Additional studies are required to gain further insight into this hypothesis.

## Materials and Methods

### Sequencing, Gene Identification and Genome Analysis

The complete genome sequencing of *Desulfovibrio piezophilus* C1TLV30^T^ was performed by Genoscope (CEA, Evry, France), and the final assembly of the C1TLV30^T^ genome contained a single replicon. The annotated sequence data were deposited in the EMBL database (accession number FO203427). The computational prediction of coding sequences (CDS) and the identification of other genome features were automatically performed by the MicroScope platform (https://www.genoscope.cns.fr/agc/microscope/home/index.php). Functional assignments were created using the MicroScope automated annotation pipeline [45] with extensive manual curation for biological processes and metabolic pathways supported by Interpro, Figfam, TMHMM and BLAST analyses. Information on additional comparative genomic analyses can be found in Text S1.

### Identification of Homologs and AA Substitution Analysis

Homologs were identified by BLAST alignment against the nr database (release of January 2012) with an E-value cutoff of 10^−3^. The AA composition of orthologous proteins was compared between *D. piezophilus* C1TLV30^T^ and *D. salexigens* DSM 2638. Orthologous proteins were defined using a reciprocal best BLASTP hit strategy with *D. salexigens* DSM 2638 as a reference genome. Additional thresholds of 70% coverage and 30% identity were added for increased stringency. Based on the reciprocal best BLAST hit, each orthologous pair was aligned using MUSCLE [46]. A systematic analysis of ungapped positions using an in-house perl script reported the numbers of conserved and substituted AAs.?For each AA, significant deviation from the expected 50∶50 ratio was determined by calculating a *χ* ^2^ with one degree of freedom.

### Cell Culture

*D. piezophilus* C1TLV30^T^ was cultured at the optimal temperature growth of 30°C, under either 0.1 MPa (atmospheric pressure, low hydrostatic pressure) or 10 MPa (high hydrostatic pressure) in the medium described by Khelaifia et al. [13]. A first run of growth at high or low hydrostatic pressure, which constituted the adaptation phase, was used to inoculate the large-scale cultures (200 mL) that were used for further proteomic and transcriptomic experiments. These large-scale cultures were inoculated at a 1∶30 ratio with the adapted cultures, and the cells were grown at high or low hydrostatic pressure until the OD_600_ nm  = 0.2. Cells from both conditions were then harvested by centrifugation at 6000 *g* for 20 min at 4°C and washed once with 20 mL of 0.1 M Tris–HCl 0.15 M NaCl buffer (pH 7.6). The pellets were subsequently used for protein and RNA extractions.

### Differential Proteomic Analysis

Proteins were extracted for analysis by either 1D gel electrophoresis for the membrane fraction or 2D gel electrophoresis for the soluble fraction as described in Text S1. After electrophoresis, the gels were silver-stained using the PlusOne Silver Staining Kit (GE Healthcare). Data were acquired from the stained gels using the Image Scanner II and Image Master 2D software from Amersham Biosciences. The intensity levels of the spots were determined by the relative spot volume of each protein compared to the normalized volume of proteins. The relative intensity of each protein spot was compared between the control and the experimental groups. An intensity ratio higher than 1.5 was considered significant, and the corresponding spot/band was selected for protein identification by MALDI-TOF mass spectrometry or LC-ESI-MS/MS. The soluble fractions were also analyzed by the DiGE methodology. Details are provided in Text S1.

### Transcript Analysis by qRT-PCR

Total RNA was isolated using the High Pure RNA Isolation Kit (Roche Diagnostics), and the cDNA was subsequently synthesized from four independent cultures. The cDNA was quantified with the SsoFast Evagreen Supermix 2X Kit (BioRad). The real-time PCR runs were carried out on a CFX96 Real-Time System using a C1000 Thermal Cycler (BioRad). The primer sequences are described in Table S7. The Relative Expression Software Tool (REST) was used to calculate the relative expression of each gene for each condition, and the 16S rRNA gene was used as a reference for normalization. The quantification was performed in duplicate or triplicate for each cDNA preparation. Details are provided in Text S1.

## Supporting Information

Figure S1 **Frequency distribution of the COG categories between** ***D. piezophilus*** **and selected** ***Desulfovibrio*** **genomes.** For each organism, COG category with a proportion away from the 2 SDs of the mean of other three organisms is considered as a significant difference and is marked by a star (red star, >2 SD; black star, <2 SD).
(TIF)

Figure S2 **Phylogenetic analysis of genes DESPIv2_11220 (A), DESPIv2_11837 (B).** The 20 best BLAST hit proteins *versus* nr were retrieved from Genbank. The phylogenetic reconstruction was achieved on the trimmed alignment using PhyML with a WAG matrix. Branch support estimated using a bootstrap of 100.
(TIF)

Figure S3 **16S RNA based Phylogenetic tree of** ***Desulfovibrio*** **strains.**
(TIF)

Table S1 **The putative genomic islands in** ***D. piezophilus*** **genome.**
(PDF)

Table S2 **List of genes encoding proteins involved in energy metabolism.**
(PDF)

Table S3 **Gene expression levels by qRT-PCR.**
(PDF)

Table S4 **The 347 genes with no homologs in sulfate reducers.**
(PDF)

Table S5 **List of the 53 COGs present in** ***D. piezophilus*** **and** ***P. profundum*** **but absent in the non-piezophilic** ***D. vulgaris*** **Hildenborough and** ***D. salexigens*** **strains.**
(PDF)

Table S6 **Average GC and GC3 content of genomes in this study and strains characteristics.**
(PDF)

Table S7 **List of oligonucleotide primers used.**
(PDF)

Table S8 **The genomic islands in** ***Desulfovibrio*** **species.**
(PDF)

Text S1 **Supplemental information on Materials and Methods.**
(DOC)
