# Supplementary figures and images for: The First Genomic and Proteomic Characterization of a Deep-Sea Sulfate Reducer: Insights into the Piezophilic Lifestyle of Desulfovibrio piezophilus
Source: PLoS One. 2013 Jan 30;8(1):e55130. doi: 10.1371/journal.pone.0055130 (PMC3559428; doi:10.1371/journal.pone.0055130)

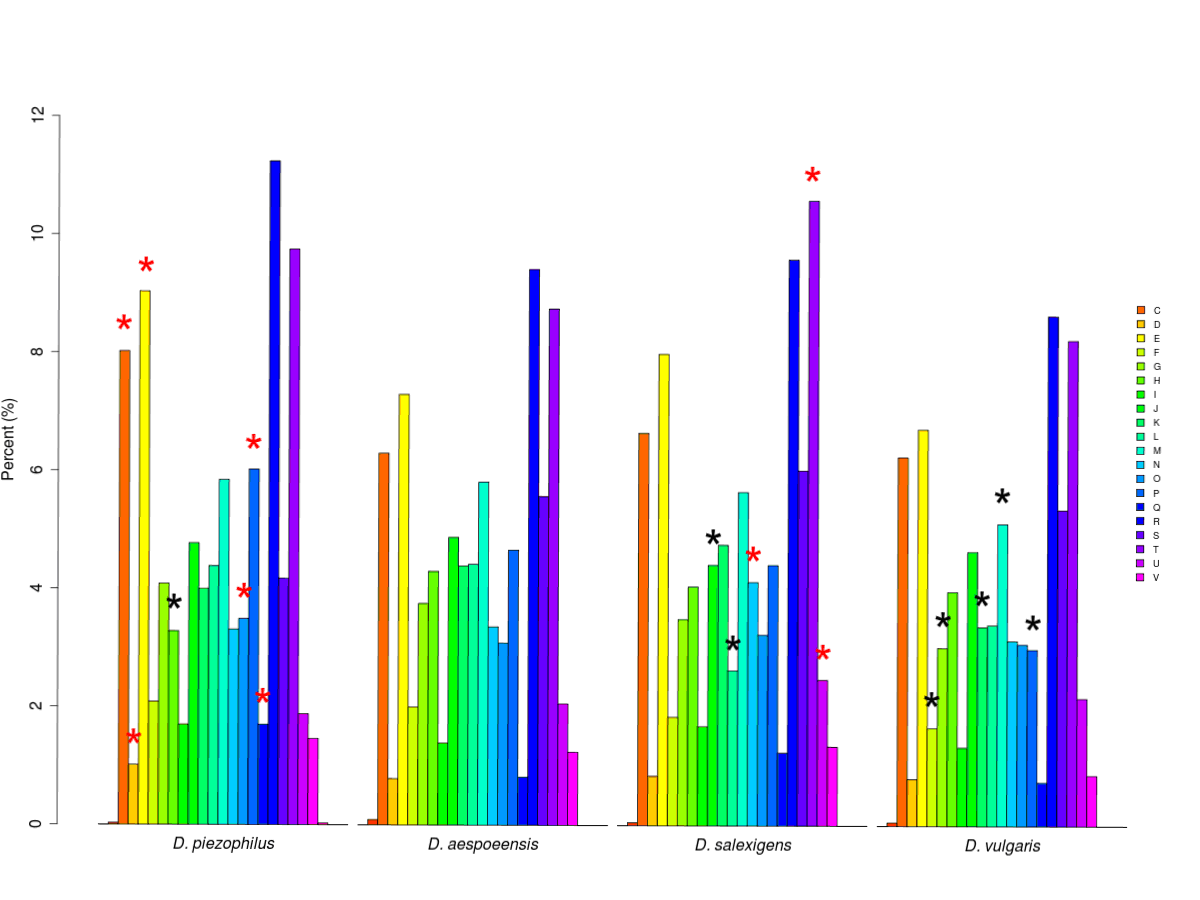

Supplement: Figure S1 — Frequency distribution of the COG categories between D. piezophilus and selected Desulfovibrio genomes. For each organism, COG category with a proportion away from the 2 SDs of the mean of other three organisms is considered as a significant difference and is marked by a star (red star, >2 SD; black star, <2 SD). (TIF) [file pone.0055130.s001.tif]

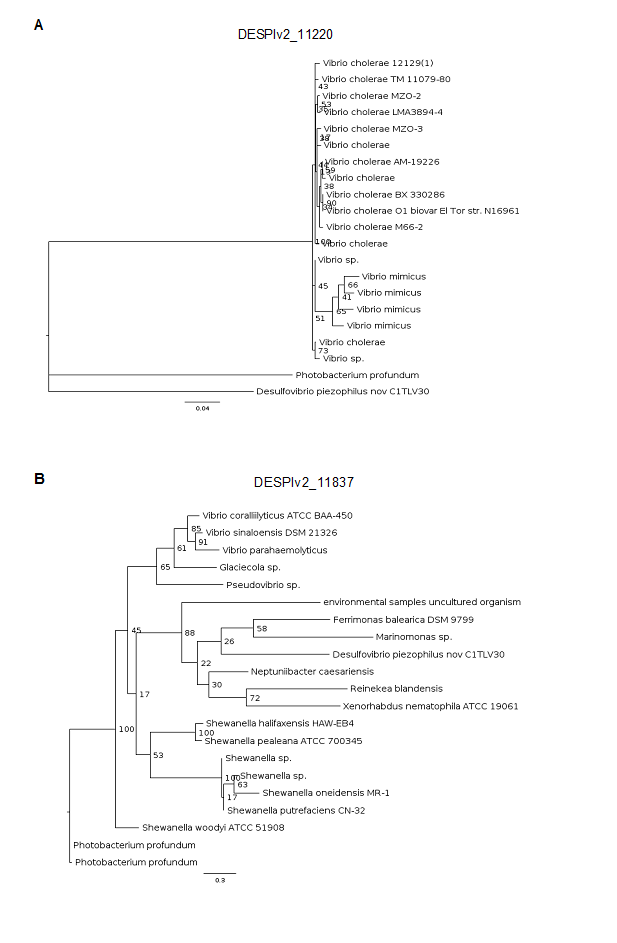

Supplement: Figure S2 — Phylogenetic analysis of genes DESPIv2_11220 (A), DESPIv2_11837 (B). The 20 best BLAST hit proteins versus nr were retrieved from Genbank. The phylogenetic reconstruction was achieved on the trimmed alignment using PhyML with a WAG matrix. Branch support estimated using a bootstrap of 100. (TIF) [file pone.0055130.s002.tif]

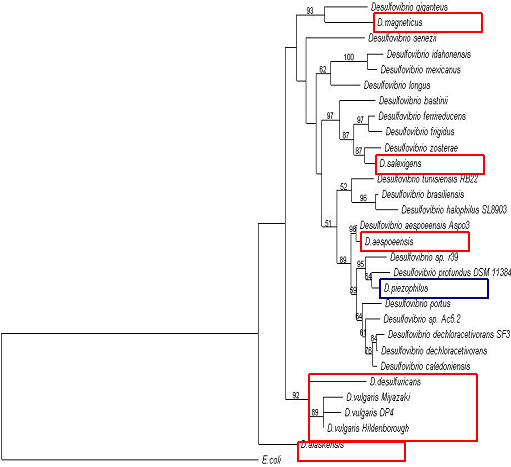

Supplement: Figure S3 — 16S RNA based Phylogenetic tree of Desulfovibrio strains. (TIF) [file pone.0055130.s003.tif]
