# Supplementary material for: The First Genomic and Proteomic Characterization of a Deep-Sea Sulfate Reducer: Insights into the Piezophilic Lifestyle of Desulfovibrio piezophilus
Source: PLoS One. 2013 Jan 30;8(1):e55130. doi: 10.1371/journal.pone.0055130 (PMC3559428; doi:10.1371/journal.pone.0055130)
Supplement: Table S1 — The putative genomic islands in D. piezophilus genome. (PDF) [file pone.0055130.s004.pdf]

Table S1. The putative genomic islands in *D. piezophilus* genome

GI	Begin	End	Length (bp)	ORFs	GC%	tRNA	transposase integrase recombinase	main function
1	313539	321767	8229	9	42.80	+	+	regulator, unknown proteins, phosphomethylpyrimidine kinase
2	473153	486599	13447	11	43.85	+	+	unknown, ion transporter, carbonhydrate metabolism
3	580740	590565	9826	15	46.76	+	+	unknown, nucleotide metabolism
4	1398185	1410524	12340	20	49.53	-	+	unknown
5	1592780	1603410	10631	19	44.76	-	+	unknown
6	2860011	2890629	30619	28	42.57	-	+	unknown, Cell envelope biogenesis, outer membrane related
7	3048200	3070115	21916	11	51.94	+	+	unknown, Energy production, outer membrane related
