# Supplementary material for: The First Genomic and Proteomic Characterization of a Deep-Sea Sulfate Reducer: Insights into the Piezophilic Lifestyle of Desulfovibrio piezophilus
Source: PLoS One. 2013 Jan 30;8(1):e55130. doi: 10.1371/journal.pone.0055130 (PMC3559428; doi:10.1371/journal.pone.0055130)
Supplement: Table S2 — List of genes encoding proteins involved in energy metabolism. (PDF) [file pone.0055130.s005.pdf]

Table S2. List of genes encoding proteins involved in energy metabolism

Cytoplasmic hydrogenases and carbon monoxide dehydrogenases

DESPIv2_11493	*EchF*	Ech hydrogenase, subunit EchF
DESPIv2_11494	*EchE*	Ech hydrogenase, subunit EchE
DESPIv2_11495	*EchD*	Ech hydrogenase, subunit EchD
DESPIv2_11496	*EchC*	Ech hydrogenase, subunit EchC
DESPIv2_11497	*EchB*	Ech hydrogenase, subunit EchB
DESPIv2_11498	*EchA*	Ech hydrogenase, subunit EchA
DESPIv2_11807		Methyl-viologen-reducing hydrogenase delta subunit
DESPIv2_20194	*cooH*	Carbon monoxide-induced hydrogenase
DESPIv2_20197	*cooL*	Carbon monoxide-induced hydrogenase, CooL subunit
DESPIv2_20189	*CooC*	Carbon monoxide dehydrogenase
DESPIv2_20190	*CooS*	Carbon monoxide dehydrogenase
DESPIv2_20191	*CooF*	FeS prootein
DESPIv2_20192	*HypA*	Hydrogenase Ni incorporation protein

Periplasmic hydrogenases

DESPIv2_11411	*hynB-1*	Periplasmic [NiFe] hydrogenase large subunit
DESPIv2_11412	*hynA-2*	Periplasmic [NiFe] hydrogenase small subunit
DESPIv2_12036	*hynA*	Periplasmic [NiFe] hydrogenase large subunit
DESPIv2_12037	*hynB*	Periplasmic [NiFe] hydrogenase small subunit 2
Others		
DESPIv2_11858	*hdrC*	Heterodisulfide reductase, subunit C
DESPIv2_11859	*hdrB*	Heterodisulfide reductase, subunit B
DESPIv2_11860	*hdrA*	Heterodisulfide reductase, subunit A
DESPIv2_11861		F420-non-reducing hydrogenase iron-sulfur subunit D
DESPIv2_11862		putative Hydrogenase
DESPIv2_11863		putative Hydrogenase
DESPIv2_11864		putative Hydrogenase

Formate dehydrogenase :

DESPIv2_11195	*fdhA*	Formate dehydrogenase subunit alpha
DESPIv2_11196	*fdhB*	Formate dehydrogenase subunit beta
DESPIv2_11197		putative Formate dehydrogenase accessory protein
DESPIv2_11198	*fdhD*	Putative protein FdHD homolog
DESPIv2_12492	*fdhB*	Formate dehydrogenase subunit beta
DESPIv2_12493 /94	*fdhA*	Formate dehydrogenase, alpha subunit
DESPIv2_20158/59	*fdhA*	Formate dehydrogenase, alpha subunit
DESPIv2_20160	*fdhB*	Formate dehydrogenase subunit beta
DESPIv2_20161	*fdhE*	Formate dehydrogenase formation protein FdhE
DESPIv2_20162	*fdhD*	Putative protein fdhD homolog

Sulfate reduction enzymes

DESPIv2_12627	*dsrA*	Sulfite reductase, dissamilatory type subunit A
DESPIv2_12628	*dsrB*	Sulfite reductase, dissamilatory type subunit B
DESPIv2_12629	*dsrD*	Sulfite reductase, dissamilatory type subunit D
DESPv2_10170	*dsrC*	Sulfite reductase, dissamilatory type subunit gamma
DESPIv2_11809	*aprA*	Adenylylsulfate reductase, alpha subunit

DESPIv2_11810	*aprB*	Adenylylsulfate reductase, beta subunit
DESPIv2_10542	*ppaC*	putative manganese-dependent inorganic pyrophosphatase
DESPIv2_11812	*sat*	Sulfate adenylyltransferase
DESPIv2_12276	*sulP*	Sulfate transporter
DESPIv2_20251	*sulP*	Sulfate transporter

Membrane-bound electron transfer complexes

DESPIv2_12499	*dsrP*	Integral membrane protein
DSEPIv2_12500	*dsrO*	Periplasmic 4Fe-4S ferredoxin (TAT substrate)
DESPIv2_12501	*dsrJ*	triheme cytochrome c
DESPIv2_12502	*dsrK*	4fe-4S clusters protein
DESPIv2_12503	*dsrM*	Inner membrane protein, binds b-type hemes
DESPIv2_11806	*qmoC*	Quinone-interacting membrane-bound oxidoreductase. b-heme and FeS protein
DESPIv2_11807	*qmoB*	Quinone-interaction membrame-bound protein oxidoreductase, flavin protein
DESPIv2_11808	*qmoA*	Quinone-interacting membrane bound oxidoreductase, Flavin protein, cytoplasmic subunit
DESPIv2_10612	*qrcD*	integral membrane protein NrfD family
DESPIv2_10613	*qrcC*	Fe-S protein
DESPIv2_10614	*qrcB*	molybdopterin-containing protein family
DESPIv2_10615	*qrcA*	pentaheme cytochrome c
DESPIv2_20227	*rnfB*	Electron transport complex, RnfB subunit
DESPIv2_20228	*rnfA*	Electron transport complex, RnfA subunit
DESPIv2_20229	*rnfE*	Electron transport complex, RnfE subunit
DESPIv2_20230	*rnfG*	Electron transport complex, RnfG subunit
DESPIv2_20231	*rnfD*	Electron transport complex, RnfD subunit
DESPIv2_20232	*rnfF*	Electron transport complex, RnfF subunit
DESPIv2_202323	*phcA*	Pentaheme cytochrome c
DESPIv2_11093	*hmcA*	Hmc complex, High-molecular-weight cytochrome c, 15 heme
DESPIv2_11092	*hmcB*	Hmc complex, subunit B, 4Fe-4S binding domain protein
DESPIv2_11091	*hmcC*	Hmc complex, subunit C
DESPIv2_11090	*hmcD*	Hmc complex, E subunit
DESPIv2_11089	*hmcF*	Hmc complex, subunit F
DESPIv2_10945	*tmcA*	Acidic cytochrome c3
DESPIv2_10946	*tmcB*	Tmc complex, sububit B 4Fe-4S domain protein
DESPIv2_10947	*tmcC*	Tmc complex, transmembrane protein
DESPIv2_10948	*tmcD*	Tmc complex, subunit D
DESPIv2_10802	*nuoN*	NADH-quinone oxidoreductase subunit N 2
DESPIv2_10803	*nuoM*	NADH-quinone oxidoreductase chain 13
DESPIv2_10804		NADH/Ubiquinone/plastoquinone (Complex I)
DESPIv2_10805		conserved protein of unknown function
DESPIv2_10806		NADH/Ubiquinone/plastoquinone (Complex I)
DESPIv2_10807		NADH-quinone oxidoreductase subunit K 1
DESPIv2_10808		NADH-ubiquinone/plastoquinone oxidoreductase chain 6 (chain J

DESPIv2_10809		4Fe-4S ferredoxin iron-sulfur binding domain protein
DESPIv2_10810		NADH-quinone oxidoreductase subunit H 1
DESPIv2_10811		NADH-quinone oxidoreductase subunit D

Cytochromes

DESPIv2_11010		Cytochrome c, 11 hemes
DESPIv2_11720	*cycA*	Type 1 Cytochrome c3
DESPIv2_11900	*Cyf*	Cytochrome c553
DESPIv2_11251	*nrfH*	cytochrome c nitrite reductase, small subunit (4 hemes)
DESPIv2_11252	*nrfA*	cytochrome c nitrite reductase, catalytic subunit (9 hemes)
DESPIv2_10723	*fd1*	Ferredoxin 1 [4Fe-4S]
DESPIv2_20267	*fd3*	Ferredoxin 3 [7Fe-8S]

Oxidases terminales

DESPIv2_11888	*cydA*	Cytochrome d oxidase, subunit 1
DESPIV2_11889	*cydB*	Cytochrome d oxidase, subunit 2
DESPIv2_11824	*cydB-like*	Cytochrome d oxidase-like
DESPIv2_11825	*cydA-like*	Cytochrome d oxidase-like
DESPIv2_11895	*coxB*	Cytochrome c oxidase, subunit II
DESPIv2_11896	*coxD*	cytochrome c oxidase subunit IV
DESPIv2_11897	*coxC*	Cytochrome c oxidase subunit III
DESPIv2_11898	*coxA*	Cytochrome c oxidase subunit 1
