# Supplementary material for: The First Genomic and Proteomic Characterization of a Deep-Sea Sulfate Reducer: Insights into the Piezophilic Lifestyle of Desulfovibrio piezophilus
Source: PLoS One. 2013 Jan 30;8(1):e55130. doi: 10.1371/journal.pone.0055130 (PMC3559428; doi:10.1371/journal.pone.0055130)
Supplement: Table S3 — Gene expression levels by qRT-PCR. (PDF) [file pone.0055130.s006.pdf]

Table S3. Gene expression levels by qRT-PCR

**Gene Accession**	**Name**	**qRT-PCR ratio (high/lowHP)**
DESPIv2_10492	Extracellular solute binding protein familly 3	< 1.70
DESPIv2_10600	GlnH	- 2.25
DESPIv2_10610	GlnH	- 2.00
DESPIv2_10845	ArgF	< 1.70
DESPIv2_11010	CytC	+ 2.08
DESPIv2_11075	DegP	<1.70
DESPIv2_11220	Unknown	+ 2.70
DESPIv2_11412	HynA	< 1.70
DESPIv2_11808	QmoA	< 1.70
DESPIv2_11809	AprA	< 1.70
DESPIv2_11824	CydB-like	+ 2.13
DESPIv2_11838	Hypothetical protein	< 1.70
DESPIv2_11888	CydA	< 1.70
DESPIv2_12106	Unknown	< 1.70
DESPIv2_12413	OmpH	- 1.80
DESPIv2_12562	HisG	<1.70
DESPIv2_20109	Unknown	+ 9.19
