# Supplementary material for: The First Genomic and Proteomic Characterization of a Deep-Sea Sulfate Reducer: Insights into the Piezophilic Lifestyle of Desulfovibrio piezophilus
Source: PLoS One. 2013 Jan 30;8(1):e55130. doi: 10.1371/journal.pone.0055130 (PMC3559428; doi:10.1371/journal.pone.0055130)
Supplement: Table S4 — The 347 genes with no homologs in sulfate reducers. (PDF) [file pone.0055130.s007.pdf]

Table S4. The 347 genes with no homologs in sulfate reducers

Gene Accession	Number of HITs matching *sulfate reducers*	Total number of HITS	Best Blast HITS annotation	COG category
DESPIv2_10028	NO HITS			
DESPIv2_10050	NO HITS			
DESPIv2_10055	NO HITS			
DESPIv2_10074	0	15	HD domain protein [delta proteobacterium NaphS2] gb|EFK07345.1| HD domain protein [delta proteobacterium NaphS2]	R
DESPIv2_10081	0	286	putative pectate lyase [Rhizobium etli CIAT 652] gb|ACE94911.1| putative pectate lyase protein [Rhizobium etli CIAT 652]	
DESPIv2_10085	0	2	hypothetical protein RHECIAT_PC0000848 [Rhizobium etli CIAT 652] gb|ACE94918.1| hypothetical protein RHECIAT_PC0000848 [Rhizobium etli CIAT 652]	
DESPIv2_10086	NO HITS			
DESPIv2_10087	NO HITS			R
DESPIv2_10108	NO HITS			
DESPIv2_10149	NO HITS			G
DESPIv2_10150	NO HITS			
DESPIv2_10164	NO HITS			
DESPIv2_10195	NO HITS			
DESPIv2_10230	NO HITS			
DESPIv2_10304	NO HITS			
DESPIv2_10308	NO HITS			
DESPIv2_10334	0	35	hypothetical protein Mbur_0327 [Methanococcoides burtonii DSM 6242] gb|ABE51323.1| Hypothetical protein Mbur_0327 [Methanococcoides burtonii DSM 6242]	C
DESPIv2_10361	NO HITS			
DESPIv2_10371	0	322	heat shock protein [Microscilla marina ATCC 23134] gb|EAY27030.1| heat shock protein [Microscilla marina ATCC 23134]	L
DESPIv2_10430	NO HITS			
DESPIv2_10454	0	> 500	transposase IS4 family protein [Rhodopseudomonas palustris DX-1] ref|YP_004109052.1| transposase IS4 family protein [Rhodopseudomonas palustris DX-1]	L
DESPIv2_10473	NO HITS			
DESPIv2_10488	NO HITS			
DESPIv2_10504	NO HITS			

DESPIv2_10507	NO HITS			
DESPIv2_10562	NO HITS			
DESPIv2_10563	NO HITS			
DESPIv2_10564	NO HITS			
DESPIv2_10565	NO HITS			
DESPIv2_10566	NO HITS			
DESPIv2_10571	0	> 500	RNA polymerase, sigma-24 subunit, ECF subfamily [Comamonas testosteroni KF-1] gb|EED67775.1| RNA polymerase, sigma-24 subunit, ECF subfamily [Comamonas testosteroni KF-1]	K
DESPIv2_10572	0	> 500	anti-FecI sigma factor, FecR [Ochrobactrum anthropi ATCC 49188] gb|ABS15484.1| anti-FecI sigma factor, FecR [Ochrobactrum anthropi ATCC 49188]	P T
DESPIv2_10574	0	> 500	Uncharacterized iron-regulated membrane protein [Oceanospirillum sp. MED92] gb|EAR61857.1| Uncharacterized iron-regulated membrane protein [Oceanospirillum sp. MED92]	S
DESPIv2_10576	NO HITS			
DESPIv2_10598	NO HITS			
DESPIv2_10620	NO HITS			
DESPIv2_10621	0	73	hypothetical protein RUMOBE_02550 [Ruminococcus obeum ATCC 29174] gb|EDM86894.1| hypothetical protein RUMOBE_02550 [Ruminococcus obeum ATCC 29174]	T
DESPIv2_10622	0	6	hypothetical protein VIB_000038 [Vibrio metschnikovii CIP 69.14] gb|EEX38574.1| hypothetical protein VIB_000038 [Vibrio metschnikovii CIP 69.14]	
DESPIv2_10623	0	3	hypothetical protein VIB_000039 [Vibrio metschnikovii CIP 69.14] gb|EEX38575.1| hypothetical protein VIB_000039 [Vibrio metschnikovii CIP 69.14]	
DESPIv2_10624	NO HITS			
DESPIv2_10625	0	> 500	serine/threonine protein kinase [Sebaldella termitidis ATCC 33386] gb|ACZ08111.1| serine/threonine protein kinase [Sebaldella termitidis ATCC 33386]	K L R T
DESPIv2_10627	NO HITS			
DESPIv2_10628	NO HITS			
DESPIv2_10631	NO HITS			
DESPIv2_10632	NO HITS			J
DESPIv2_10633	0	> 500	chromosome partitioning ATPase-like protein [Shewanella baltica OS155] ref|YP_001355411.1| chromosome partitioning ATPase-like protein [Shewanella baltica OS185]	D L
DESPIv2_10634	NO HITS			
DESPIv2_10637	0	1	hypothetical protein Moth_0713 [Moorella thermoacetica ATCC 39073] gb|ABC19031.1| conserved hypothetical protein [Moorella thermoacetica ATCC 39073]	
DESPIv2_10641	NO HITS			
DESPIv2_10642	0	103	hypothetical protein Dace_1994 [Desulfuromonas acetoxidans DSM 684] gb|EAT16742.1| hypothetical protein Dace_1994 [Desulfuromonas acetoxidans DSM 684]	

DESPIv2_10644	NO HITS			V
DESPIv2_10646	NO HITS			
DESPIv2_10687	NO HITS			
DESPIv2_10760	NO HITS			
DESPIv2_10775	NO HITS			
DESPIv2_10801	NO HITS			
DESPIv2_10818	0	> 500	transposase IS4 family protein [Rhodopseudomonas palustris DX-1] ref|YP_004109052.1| transposase IS4 family protein [Rhodopseudomonas palustris DX-1]	L
DESPIv2_10859	NO HITS			
DESPIv2_10879	NO HITS			
DESPIv2_10882	NO HITS			
DESPIv2_10887	0	191	hypothetical protein Isop_3507 [Isosphaera pallida ATCC 43644] gb|ADV64064.1| hypothetical protein Isop_3507 [Isosphaera pallida ATCC 43644]	H R
DESPIv2_10889	NO HITS			
DESPIv2_10890	NO HITS			
DESPIv2_10891	0	1	site-specific recombinase, phage integrase family [delta proteobacterium NaphS2] gb|EFK09300.1| site-specific recombinase, phage integrase family [delta proteobacterium NaphS2]	L
DESPIv2_10916	NO HITS			
DESPIv2_10917	NO HITS			
DESPIv2_10918	NO HITS			
DESPIv2_10921	NO HITS			
DESPIv2_10922	0	> 500	transposase IS4 family protein [Rhodopseudomonas palustris DX-1] ref|YP_004109052.1| transposase IS4 family protein [Rhodopseudomonas palustris DX-1]	L
DESPIv2_10925	NO HITS			
DESPIv2_10928	NO HITS			
DESPIv2_10940	NO HITS			
DESPIv2_10952	NO HITS			
DESPIv2_10984	NO HITS			
DESPIv2_11000	NO HITS			
DESPIv2_11010	0	51	cytochrome c family protein [Photobacterium profundum 3TCK] gb|EAS43043.1| cytochrome c family protein [Photobacterium profundum 3TCK]	
DESPIv2_11017	NO HITS			
DESPIv2_11030	0	6	orfI [Pseudomonas aeruginosa]	
DESPIv2_11034	NO HITS			
DESPIv2_11037	NO HITS			
DESPIv2_11062	NO HITS			

DESPIv2_11069	0	77	hypothetical protein Bamb_4252 [Burkholderia ambifaria AMMD] gb|ABI89805.1| conserved hypothetical protein [Burkholderia ambifaria AMMD]	
DESPIv2_11073	NO HITS			
DESPIv2_11094	NO HITS			
DESPIv2_11111	NO HITS			
DESPIv2_11112	NO HITS			
DESPIv2_11113	NO HITS			
DESPIv2_11114	NO HITS			
DESPIv2_11120	NO HITS			L
DESPIv2_11122	NO HITS			
DESPIv2_11124	NO HITS			
DESPIv2_11127	0	162	esterase/lipase [Agrobacterium radiobacter K84] gb|ACM30148.1| esterase/lipase [Agrobacterium radiobacter K84]	I
DESPIv2_11135	0	> 500	Carboxymuconolactone decarboxylase [Yersinia bercovieri ATCC 43970] gb|EEQ07835.1| Carboxymuconolactone decarboxylase [Yersinia bercovieri ATCC 43970]	S
DESPIv2_11142	NO HITS			
DESPIv2_11143	NO HITS			
DESPIv2_11151	NO HITS			
DESPIv2_11169	NO HITS			
DESPIv2_11170	NO HITS			
DESPIv2_11180| yicE	0	> 500	xanthine permease [Spirochaeta smaragdinae DSM 11293] gb|ADK82056.1| xanthine permease [Spirochaeta smaragdinae DSM 11293]	F
DESPIv2_11193	NO HITS			
DESPIv2_11194	0	2	hypothetical protein RB2150_05173 [Rhodobacterales bacterium HTCC2150] gb|EBA03864.1| hypothetical protein RB2150_05173 [Rhodobacterales bacterium HTCC2150]	R
DESPIv2_11206	NO HITS			
DESPIv2_11207	0	72	Spore coat protein CotH [Dethiobacter alkaliphilus AHT 1] gb|EEG78404.1| Spore coat protein CotH [Dethiobacter alkaliphilus AHT 1]	
DESPIv2_11208	0	8	hypothetical protein P9211_07661 [Prochlorococcus marinus str. MIT 9211] gb|ABX08697.1| Hypothetical protein P9211_07661 [Prochlorococcus marinus str. MIT 9211]	E G R
DESPIv2_11209	0	13	hypothetical protein P9211_07681 [Prochlorococcus marinus str. MIT 9211] gb|ABX08699.1| Hypothetical protein P9211_07681 [Prochlorococcus marinus str. MIT 9211]	P
DESPIv2_11210	0	46	Dihydrodipicolinate synthase/N-acetylneuraminate lyase [Yersinia rohdei ATCC 43380] gb|EEQ04477.1| Dihydrodipicolinate synthase/N-acetylneuraminate lyase [Yersinia rohdei ATCC 43380]	E M
DESPIv2_11211	NO HITS			
DESPIv2_11212	NO HITS			
DESPIv2_11214	NO HITS			

DESPIv2_11215	0	> 500	multidrug efflux pump AmrA [Achromobacter piechaudii ATCC 43553] gb|EFF74915.1| multidrug efflux pump AmrA [Achromobacter piechaudii ATCC 43553]	L M
DESPIv2_11220	0	> 500	putative iron(III) compound receptor [Photobacterium profundum SS9] emb|CAG19977.1| putative iron(III) compound receptor [Photobacterium profundum SS9]	P
DESPIv2_11222	0	> 500	iron(III) ABC transporter, periplasmic iron-compound-binding protein [Vibrio cholerae O1 biovar El Tor str. N16961] ref|ZP_01677328.1|	P
DESPIv2_11223	0	> 500	iron-hydroxamate transporter permease subunit [Photobacterium profundum SS9] emb|CAG19974.1| hypothetical iron(III) ABC transporter, permease protein [Photobacterium profundum SS9]	P
DESPIv2_11224	NO HITS			
DESPIv2_11228	NO HITS			
DESPIv2_11233	0	1	methyltransferase family [Actinomyces sp. oral taxon 171 str. F0337] gb|EFW27719.1| methyltransferase family [Actinomyces sp. oral taxon 171 str. F0337]	Q
DESPIv2_11234	0	154	hypothetical protein [Fischerella sp. MV11]	E
DESPIv2_11235	0	26	hypothetical protein [Fischerella sp. MV11]	E
DESPIv2_11236	0	126	AMP-binding enzyme [Bilophila wadsworthia 3_1_6] gb|EFV44812.1| AMP-binding enzyme [Bilophila wadsworthia 3_1_6]	Q
DESPIv2_11237	0	17	hypothetical protein HMPREF1016_00246 [Bacteroides eggerthii 1_2_48FAA] gb|EFV31506.1| hypothetical protein HMPREF1016_00246 [Bacteroides eggerthii 1_2_48FAA]	O
DESPIv2_11245	0	> 500	AMP-binding enzyme [Bilophila wadsworthia 3_1_6] gb|EFV44812.1| AMP-binding enzyme [Bilophila wadsworthia 3_1_6]	I Q
DESPIv2_11250	NO HITS			O
DESPIv2_11253	NO HITS			
DESPIv2_11254	0	2	lysM domain protein [Brevundimonas diminuta ATCC 11568] gb|EGF94156.1| lysM domain protein [Brevundimonas diminuta ATCC 11568]	
DESPIv2_11255	0	17	hypothetical protein MucpaDRAFT_0488 [Mucilaginibacter paludis DSM 18603] gb|EFQ74703.1| hypothetical protein MucpaDRAFT_0488 [Mucilaginibacter paludis DSM 18603]	
DESPIv2_11256	NO HITS			
DESPIv2_11257	NO HITS			
DESPIv2_11258	NO HITS			
DESPIv2_11261	0	499	Lysozyme [Methylobacter tundripaludum SV96] gb|EFO04657.1| Lysozyme [Methylobacter tundripaludum SV96]	R
DESPIv2_11262	0	22	hypothetical protein PcarcW_14964 [Pectobacterium carotovorum subsp. carotovorum WPP14]	
DESPIv2_11265	NO HITS			
DESPIv2_11266	NO HITS			E
DESPIv2_11267	0	15	hypothetical protein STAUR_8252 [Stigmatella aurantiaca DW4/3-1] gb|ADO76007.1| uncharacterized protein [Stigmatella aurantiaca DW4/3-1]	
DESPIv2_11268	NO HITS			

DESPIv2_11270	NO HITS			
DESPIv2_11272	0	18	hypothetical protein MucpaDRAFT_0488 [Mucilaginibacter paludis DSM 18603] gb|EFQ74703.1| hypothetical protein MucpaDRAFT_0488 [Mucilaginibacter paludis DSM 18603]	
DESPIv2_11273	NO HITS			
DESPIv2_11274	NO HITS			
DESPIv2_11277	NO HITS			
DESPIv2_11278	NO HITS			
DESPIv2_11279	0	179	RTX toxin-activating lysine-acyltransferase [Bradyrhizobium sp. ORS278] emb|CAL80517.1| RTX toxin-activating lysine-acyltransferase (type I secretion system) [Bradyrhizobium sp. ORS278]	O
DESPIv2_11280	NO HITS			
DESPIv2_11282	NO HITS			
DESPIv2_11284	NO HITS			
DESPIv2_11286	NO HITS			
DESPIv2_11301	NO HITS			
DESPIv2_11306	NO HITS			
DESPIv2_11322	NO HITS			
DESPIv2_11329	NO HITS			N T
DESPIv2_11336	NO HITS			
DESPIv2_11337	NO HITS			E P
DESPIv2_11338	0	1	hypothetical protein SCHCODRAFT_53948 [Schizophyllum commune H4-8] gb|EFI98062.1| hypothetical protein SCHCODRAFT_53948 [Schizophyllum commune H4-8]	
DESPIv2_11339	NO HITS			
DESPIv2_11342	0	60	hypothetical protein IMCC1989_529 [gamma proteobacterium IMCC1989] gb|EGG93938.1| hypothetical protein IMCC1989_529 [gamma proteobacterium IMCC1989]	S
DESPIv2_11343	NO HITS			
DESPIv2_11348	NO HITS			
DESPIv2_11349	NO HITS			
DESPIv2_11351	0	18	UDP-GlcNAc-specific C4,6 dehydratase/C5 epimerase [Campylobacterales bacterium GD 1] gb|EDZ62373.1| UDP-GlcNAc-specific C4,6 dehydratase/C5 epimerase [Campylobacterales bacterium GD 1]	G M
DESPIv2_11352	NO HITS			
DESPIv2_11357	0	7	NUDIX hydrolase [Nitrobacter hamburgensis X14] gb|ABE62029.1| NUDIX hydrolase [Nitrobacter hamburgensis X14]	
DESPIv2_11382	0	36	hypothetical protein LA_1823 [Leptospira interrogans serovar Lai str. 56601] gb|AAN49022.1| hypothetical protein LA_1823 [Leptospira interrogans serovar Lai str. 56601]	
DESPIv2_11424	0	120	GCN5-related N-acetyltransferase [Clostridium beijerinckii NCIMB 8052] gb|ABR35953.1| GCN5-related N-acetyltransferase [Clostridium beijerinckii NCIMB 8052]	K R
DESPIv2_11427	NO HITS			

DESPIv2_11488	NO HITS			
DESPIv2_11492	NO HITS			
DESPIv2_11501	NO HITS			
DESPIv2_11508	NO HITS			
DESPIv2_11521	0	3	insecticidal toxin SepC/TccC [Listeria seeligeri FSL S4-171]	
DESPIv2_11522	0	12	conserved hypothetical protein [Oscillatoria sp. PCC 6506] emb|CBN58573.1| conserved hypothetical protein [Oscillatoria sp. PCC 6506]	
DESPIv2_11523	0	1	hypothetical protein TR2A62_0703 [Thalassiobium sp. R2A62] gb|EET47116.1| hypothetical protein TR2A62_0703 [Thalassiobium sp. R2A62]	
DESPIv2_11524	0	43	hypothetical protein ymoll0001_41190 [Yersinia mollaretii ATCC 43969] gb|EEQ08594.1| hypothetical protein ymoll0001_41190 [Yersinia mollaretii ATCC 43969]	U
DESPIv2_11525	NO HITS			
DESPIv2_11526	0	5	conserved hypothetical protein [Prevotella salivae DSM 15606] gb|EFV05732.1| conserved hypothetical protein [Prevotella salivae DSM 15606]	U
DESPIv2_11527	NO HITS			
DESPIv2_11528	0	5	hypothetical protein Caci_3518 [Catenulispora acidiphila DSM 44928] gb|ACU72424.1| hypothetical protein Caci_3518 [Catenulispora acidiphila DSM 44928]	
DESPIv2_11529	0	25	colicin-D domain protein [Escherichia coli 1180]	U
DESPIv2_11530	NO HITS			
DESPIv2_11531	NO HITS			
DESPIv2_11532	0	17	insecticidal toxin SepC/TccC [Listeria seeligeri FSL S4-171]	U
DESPIv2_11533	0	16	conserved hypothetical protein [Oscillatoria sp. PCC 6506] emb|CBN58573.1| conserved hypothetical protein [Oscillatoria sp. PCC 6506]	
DESPIv2_11536	NO HITS			
DESPIv2_11537	NO HITS			
DESPIv2_11538	NO HITS			
DESPIv2_11539	NO HITS			
DESPIv2_11554	NO HITS			
DESPIv2_11561	NO HITS			
DESPIv2_11568| glpE	NO HITS			
DESPIv2_11579	NO HITS			
DESPIv2_11604	0	1	hypothetical protein ALOHA_HF4000APKG2M17ctg1g16 [uncultured marine microorganism HF4000_APKG2M17]	
DESPIv2_11615	NO HITS			
DESPIv2_11616	NO HITS			

DESPIv2_11617	0	> 500	extracellular solute-binding protein, family 1 [Pseudovibrio sp. JE062] gb|EEA94584.1| extracellular solute-binding protein, family 1 [Pseudovibrio sp. JE062]	G
DESPIv2_11619	NO HITS			
DESPIv2_11621	NO HITS			
DESPIv2_11627	NO HITS			
DESPIv2_11644	NO HITS			
DESPIv2_11655	0	222	hemolysin-type calcium-binding region [Azospirillum sp. B510] dbj|BAI75749.1| hemolysin-type calcium-binding region [Azospirillum sp. B510]	U W
DESPIv2_11667	NO HITS			
DESPIv2_11681	NO HITS			
DESPIv2_11684	NO HITS			
DESPIv2_11722	NO HITS			
DESPIv2_11751	0	3	hypothetical protein Mmc1_0738 [Magnetococcus sp. MC-1] gb|ABK43259.1| hypothetical protein Mmc1_0738 [Magnetococcus sp. MC-1]	
DESPIv2_11763	0	8	putative transmembrane protein [Xanthobacter autotrophicus Py2] gb|ABS67484.1| putative transmembrane protein [Xanthobacter autotrophicus Py2]	
DESPIv2_11769	NO HITS			
DESPIv2_11811	NO HITS			
DESPIv2_11831	NO HITS			
DESPIv2_11835	NO HITS			
DESPIv2_11837	0	> 500	hypothetical transcriptional regulator, ArsR family protein [Oceanospirillum sp. MED92] gb|EAR62696.1| hypothetical transcriptional regulator, ArsR family protein [Oceanospirillum sp. MED92]	K
DESPIv2_11838	0	54	hypothetical protein Amet_4000 [Alkaliphilus metalliredigens QYMF] gb|ABR50082.1| hypothetical protein Amet_4000 [Alkaliphilus metalliredigens QYMF]	
DESPIv2_11848	NO HITS			
DESPIv2_11853	NO HITS			
DESPIv2_11881	0	> 500	cysteine desulphurase-like protein [Candidatus Koribacter versatilis Ellin345] gb|ABF39641.1| Cysteine desulfurase related, unknown function [Candidatus Koribacter versatilis Ellin345]	E R
DESPIv2_11882	0	> 500	GntR family transcriptional regulator [Thermoanaerobacter sp. X514] ref|ZP_07131216.1| transcriptional regulator, GntR family [Thermoanaerobacter sp. X561]	K
DESPIv2_11892	NO HITS			
DESPIv2_11902	0	3	Conserved hypothetical protein, membrane [Salinibacter ruber M8] emb|CBH26002.1| Conserved hypothetical protein, membrane [Salinibacter ruber M8]	
DESPIv2_11963	NO HITS			E P
DESPIv2_11965	NO HITS			
DESPIv2_11992	NO HITS			

DESPIv2_12041	NO HITS			
DESPIv2_12042	NO HITS			T
DESPIv2_12043	NO HITS			
DESPIv2_12044	NO HITS			
DESPIv2_12045	0	> 500	serine/threonine protein kinase [Thermobispora bispora DSM 43833] gb|ADG89546.1| serine/threonine protein kinase [Thermobispora bispora DSM 43833]	K L R T
DESPIv2_12046	NO HITS			
DESPIv2_12058	NO HITS			
DESPIv2_12063	NO HITS			
DESPIv2_12071	0	37	probable glycosyl transferase [Arthrospira platensis NIES-39]	M
DESPIv2_12072	0	3	hypothetical protein cce_0514 [Cyanothece sp. ATCC 51142] gb|ACB49865.1| hypothetical protein cce_0514 [Cyanothece sp. ATCC 51142]	
DESPIv2_12074	NO HITS			I
DESPIv2_12082	0	> 500	radical SAM protein [Clostridium cellulolyticum H10] gb|ACL75407.1| Radical SAM domain protein [Clostridium cellulolyticum H10]	J
DESPIv2_12091	0	1	hypothetical protein Plut_1185 [Chlorobium luteolum DSM 273] gb|ABB24047.1| conserved hypothetical protein [Chlorobium luteolum DSM 273]	
DESPIv2_12098	0	229	transposon, transposition helper protein C, putative [Acidithiobacillus ferrooxidans ATCC 23270] gb|ACK78888.1| transposon, transposition helper protein C, putative [Acidithiobacillus ferrooxidans ATCC 23270]	O
DESPIv2_12099	0	25	hypothetical protein Lferr_2127 [Acidithiobacillus ferrooxidans ATCC 53993] gb|ACH84333.1| conserved hypothetical protein [Acidithiobacillus ferrooxidans ATCC 53993]	L
DESPIv2_12100	NO HITS			
DESPIv2_12101	0	147	hypothetical protein Psyr_0097 [Pseudomonas syringae pv. syringae B728a] gb|AAY35171.1| hypothetical protein Psyr_0097 [Pseudomonas syringae pv. syringae B728a]	
DESPIv2_12103	0	113	hypothetical protein Psyr_0099 [Pseudomonas syringae pv. syringae B728a] gb|AAY35173.1| Conserved hypothetical protein [Pseudomonas syringae pv. syringae B728a]	
DESPIv2_12104	0	251	hypothetical protein Mmc1_2716 [Magnetococcus sp. MC-1] gb|ABK45209.1| conserved hypothetical protein [Magnetococcus sp. MC-1]	K
DESPIv2_12105	0	4	hypothetical protein Noc_0705 [Nitrosococcus oceani ATCC 19707] ref|ZP_05048781.1	
DESPIv2_12106	0	5	hypothetical protein Noc_0706 [Nitrosococcus oceani ATCC 19707] gb|ABA57220.1| hypothetical protein Noc_0706 [Nitrosococcus oceani ATCC 19707]	E
DESPIv2_12107	0	4	hypothetical protein Noc_0707 [Nitrosococcus oceani ATCC 19707] ref|ZP_05048634.1	D L
DESPIv2_12108	0	3	hypothetical protein Bcep18194_C6534 [Burkholderia sp. 383] gb|ABB05585.1| hypothetical protein Bcep18194_C6534 [Burkholderia sp. 383]	
DESPIv2_12109	NO HITS			
DESPIv2_12110	NO HITS			

DESPIv2_12112	0	7	hypothetical protein CPS_2405 [Colwellia psychrerythraea 34H] gb|AAZ26288.1| hypothetical protein CPS_2405 [Colwellia psychrerythraea 34H]	
DESPIv2_12113	0	16	hypothetical protein Rpic12D_0210 [Ralstonia pickettii 12D] gb|ACS61519.1| conserved hypothetical protein [Ralstonia pickettii 12D]	
DESPIv2_12123	NO HITS			
DESPIv2_12127	NO HITS			
DESPIv2_12138	NO HITS			
DESPIv2_12139	NO HITS			
DESPIv2_12147	NO HITS			
DESPIv2_12181	NO HITS			
DESPIv2_12200	NO HITS			
DESPIv2_12211	NO HITS			
DESPIv2_12226	NO HITS			
DESPIv2_12228	0	82	putative ribonuclease inhibitor YrdF [Bacillus licheniformis ATCC 14580] ref|YP_093232.1| YrdF [Bacillus licheniformis ATCC 14580]	
DESPIv2_12233	NO HITS			
DESPIv2_12235	NO HITS			
DESPIv2_12236	NO HITS			
DESPIv2_12237	NO HITS			G
DESPIv2_12238	NO HITS			
DESPIv2_12242	NO HITS			
DESPIv2_12243	NO HITS			
DESPIv2_12250	0	4	hypothetical protein VP10329_05657 [Vibrio parahaemolyticus 10329]	
DESPIv2_12252	NO HITS			
DESPIv2_12255	NO HITS			
DESPIv2_12256	NO HITS			
DESPIv2_12257	NO HITS			
DESPIv2_12259	NO HITS			
DESPIv2_12295	0	> 500	type III polyketide synthase [Azotobacter vinelandii DJ] gb|ACO79121.1| Type III polyketide synthase [Azotobacter vinelandii DJ]	Q
DESPIv2_12306	NO HITS			
DESPIv2_12309	NO HITS			
DESPIv2_12320	NO HITS			
DESPIv2_12346	NO HITS			
DESPIv2_12367	NO HITS			
DESPIv2_12370	NO HITS			

DESPIv2_12374	NO HITS			
DESPIv2_12428	NO HITS			
DESPIv2_12449	NO HITS			
DESPIv2_12465	NO HITS			
DESPIv2_12547	NO HITS			
DESPIv2_12565	NO HITS			
DESPIv2_12571	NO HITS			
DESPIv2_12580	NO HITS			
DESPIv2_12586	NO HITS			
DESPIv2_12599	0	1	putative integron gene cassette protein [uncultured bacterium]	
DESPIv2_12611	NO HITS			
DESPIv2_12616	NO HITS			
DESPIv2_12617	NO HITS			
DESPIv2_12619	NO HITS			
DESPIv2_12630	NO HITS			
DESPIv2_12650	NO HITS			
DESPIv2_12671	NO HITS			
DESPIv2_12677	0	2	hypothetical protein P9215_14621 [Prochlorococcus marinus str. MIT 9215] gb|ABV51075.1| Hypothetical protein P9215_14621 [Prochlorococcus marinus str. MIT 9215]	
DESPIv2_12679	0	43	hypothetical protein Plim_4022 [Planctomyces limnophilus DSM 3776] gb|ADG69833.1| conserved hypothetical protein [Planctomyces limnophilus DSM 3776]	
DESPIv2_12684	NO HITS			
DESPIv2_12685	NO HITS			
DESPIv2_12691	0	1	hypothetical protein Namu_4448 [Nakamurella multipartita DSM 44233] gb|ACV80734.1| conserved hypothetical protein [Nakamurella multipartita DSM 44233]	N T
DESPIv2_12693	0	3	hypothetical protein RBWH47_00894 [Rhodopirellula baltica WH47]	
DESPIv2_12700| yhcX	0	> 500	hydrolase, carbon-nitrogen family [Verrucomicrobiae bacterium DG1235] gb|EDY85244.1| hydrolase, carbon-nitrogen family [Verrucomicrobiae bacterium DG1235]	K M R
DESPIv2_12701	NO HITS			
DESPIv2_12781	NO HITS			
DESPIv2_12785	NO HITS			
DESPIv2_12793	NO HITS			
DESPIv2_12817	NO HITS			
DESPIv2_12821	NO HITS			
DESPIv2_20009	NO HITS			

DESPIv2_20010	0	38	hypothetical protein NAEGRDRAFT_59370 [Naegleria gruberi] gb|EFC38981.1| hypothetical protein NAEGRDRAFT_59370 [Naegleria gruberi]	
DESPIv2_20011	NO HITS			
DESPIv2_20028	0	302	Tat pathway signal sequence domain protein [delta proteobacterium NaphS2] gb|EFK07501.1| Tat pathway signal sequence domain protein [delta proteobacterium NaphS2]	R
DESPIv2_20045	NO HITS			
DESPIv2_20056	NO HITS			
DESPIv2_20066	NO HITS			
DESPIv2_20080	NO HITS			
DESPIv2_20094	NO HITS			
DESPIv2_20095	NO HITS			
DESPIv2_20116	NO HITS			
DESPIv2_20118	NO HITS			
DESPIv2_20119	NO HITS			
DESPIv2_20120	NO HITS			R
DESPIv2_20122	NO HITS			G O
DESPIv2_20130	NO HITS			
DESPIv2_20131	NO HITS			
DESPIv2_20134	NO HITS			
DESPIv2_20154	NO HITS			
DESPIv2_20181	NO HITS			
DESPIv2_20193	NO HITS			
DESPIv2_20201	NO HITS			
DESPIv2_20207	NO HITS			
DESPIv2_20208	0	4	putative lipoprotein [Leptospira biflexa serovar Patoc strain 'Patoc 1 (Ames)'] ref|YP_001964454.1| hypothetical protein LEPBI_II0052 [Leptospira biflexa serovar Patoc strain 'Patoc 1 (Paris)']	
DESPIv2_20221	NO HITS			C
DESPIv2_20222	NO HITS			
DESPIv2_20225	0	12	Leucine-rich repeat:Na-Ca exchanger/integrin-beta4:Putative Ig [Crocosphaera watsonii WH 8501] gb|EAM49003.1| Leucine-rich repeat:Na-Ca exchanger/integrin-beta4:Putative Ig [Crocosphaera watsonii WH 8501]	
DESPIv2_20226	NO HITS			
DESPIv2_20244	0	1	Cupin 2 conserved barrel domain protein [Denitrovibrio acetiphilus DSM 12809] gb|ADD67841.1| Cupin 2 conserved barrel domain protein [Denitrovibrio acetiphilus DSM 12809]	
DESPIv2_20252	NO HITS			
DESPIv2_20332	NO HITS			
DESPIv2_20342	0	2	hypothetical protein RBWH47_00894 [Rhodopirellula baltica WH47]	

DESPIv2_20356	0	> 500	MinD/ParA family ATPase [Laribacter hongkongensis] gb|ABV90267.1| putative MinD/ParA family ATPase [Laribacter hongkongensis]	D
DESPIv2_20357	0	1	hypothetical protein LHK_00895 [Laribacter hongkongensis HLHK9] gb|ACO73888.1| hypothetical protein LHK_00895 [Laribacter hongkongensis HLHK9]	
DESPIv2_20398	NO HITS			
DESPIv2_20402	NO HITS			
DESPIv2_20403	NO HITS			
DESPIv2_20404	NO HITS			
DESPIv2_20448	NO HITS			
DESPIv2_20453	NO HITS			
DESPIv2_20456| secE	NO HITS			U
DESPIv2_20532	NO HITS			

Clusters are in grey shaded
