# Supplementary material for: The First Genomic and Proteomic Characterization of a Deep-Sea Sulfate Reducer: Insights into the Piezophilic Lifestyle of Desulfovibrio piezophilus
Source: PLoS One. 2013 Jan 30;8(1):e55130. doi: 10.1371/journal.pone.0055130 (PMC3559428; doi:10.1371/journal.pone.0055130)
Supplement: Table S5 — List of the 53 COGs present in D. piezophilus and P. profundum but absent in the non-piezophilic D. vulgaris Hildenborough and D. salexigens strains. (PDF) [file pone.0055130.s008.pdf]

Table S5. List of the 53 COGs present in *D. piezophilus* and *P.profundum* but absent in the non-piezophilic *D. vulgaris* Hildenborough and *D. salexigens* strains

COGs	Category	Function
COG2080	C	Aerobic-type carbon monoxide dehydrogenase, small subunit CoxS/CutS homologs
COG2010	C	Cytochrome c, mono- and diheme variants
COG1071	C	Pyruvate/2-oxoglutarate dehydrogenase complex, dehydrogenase (E1) component, alpha subunit
COG0022	C	Pyruvate/2-oxoglutarate dehydrogenase complex, dehydrogenase (E1) component, beta subunit
COG0508	C	Pyruvate/2-oxoglutarate dehydrogenase complex, dihydrolipoamide acyltransferase (E2) component
COG0160	E	4-aminobutyrate aminotransferase and related aminotransferases
COG3075	E	Anaerobic glycerol-3-phosphate dehydrogenase
COG2303	E	Choline dehydrogenase and related flavoproteins
COG2873	E	O-acetylhomoserine sulfhydrylase
COG0520	E	Selenocysteine lyase
COG1171	E	Threonine dehydratase
COG1305	E	Transglutaminase-like enzymes, putative cysteine proteases
COG4630	F	Xanthine dehydrogenase, iron-sulfur cluster and FAD-binding subunit A
COG0364	G	Glucose-6-phosphate 1-dehydrogenase
COG3408	G	Glycogen debranching enzyme
COG3840	H	ABC-type thiamine transport system, ATPase component
COG0314	H	Molybdopterin converting factor, large subunit
COG0746	H	Molybdopterin-guanine dinucleotide biosynthesis protein A
COG0245	I	2C-methyl-D-erythritol 2,4-cyclodiphosphate synthase
COG0825	I	Acetyl-CoA carboxylase alpha subunit
COG1443	I	Isopentenyldiphosphate isomerase
COG2378	K	Predicted transcriptional regulator
COG4335	L	DNA alkylation repair enzyme
COG1943	L	Transposase and inactivated derivatives
COG3209	M	Rhs family protein
COG0615	MI	Cytidylyltransferase
COG0544	O	FKBP-type peptidyl-prolyl cis-trans isomerase (trigger factor)
COG3653	Q	N-acyl-D-aspartate/D-glutamate deacylase
COG1020	Q	Non-ribosomal peptide synthetase modules and related proteins
COG3315	Q	O-Methyltransferase involved in polyketide biosynthesis
COG4664	Q	TRAP-type mannitol/chloroaromatic compound transport system, large permease component

COG4663	Q	TRAP-type mannitol/chloroaromatic compound transport system, periplasmic component
COG4665	Q	TRAP-type mannitol/chloroaromatic compound transport system, small permease component
COG3302	R	DMSO reductase anchor subunit
COG1075	R	Predicted acetyltransferases and hydrolases with the alpha/beta hydrolase fold
COG1342	R	Predicted DNA-binding proteins
COG2333	R	Predicted hydrolase (metallo-beta-lactamase superfamily)
COG0628	R	Predicted permease
COG0679	R	Predicted permease
COG0637	R	Predicted phosphatase/phosphohexomutase
COG1694	R	Predicted pyrophosphatase
COG2119	S	Predicted membrane protein
COG2314	S	Predicted membrane protein
COG0316	S	Uncharacterized conserved protein
COG0391	S	Uncharacterized conserved protein
COG4895	S	Uncharacterized conserved protein
COG3016	S	Uncharacterized iron-regulated protein
COG3123	S	Uncharacterized protein conserved in bacteria
COG4222	S	Uncharacterized protein conserved in bacteria
COG2062	T	Phosphohistidine phosphatase SixA
COG4566	T	Response regulator
COG4753	T	Response regulator containing CheY-like receiver domain and AraC-type DNA-binding domain
COG1566	V	Multidrug resistance efflux pump
