# Supplementary material for: The First Genomic and Proteomic Characterization of a Deep-Sea Sulfate Reducer: Insights into the Piezophilic Lifestyle of Desulfovibrio piezophilus
Source: PLoS One. 2013 Jan 30;8(1):e55130. doi: 10.1371/journal.pone.0055130 (PMC3559428; doi:10.1371/journal.pone.0055130)
Supplement: Table S6 — Average GC and GC3 content of genomes in this study and strains characteristics. (PDF) [file pone.0055130.s009.pdf]

Table S6. Average GC and GC3 content of genomes in this study and strains characteristics

Organism	Taxonomic affiliation	GC%	GC3%	OGT[1]	Habitat[2]
Desulfovibrio piezophilus	*Desulfovibrio*	49.9	51.9	30	Deep sea
Desulfovibrio aespoeensis	*Desulfovibrio*	62.5	82.9	25-30	Animal intestinal microflora, Fresh water, Mud, Sea water, Soil
Desulfovibrio alaskensis	*Desulfovibrio*	57.8	65.3	37	Oil well
Desulfovibrio desulfuricans	*Desulfovibrio*	58.0	66.3	37(30 ?)	Fresh water, Rumen, Host
Desulfovibrio magneticus	*Desulfovibrio*	62.7	78.9	25	Fresh water
Desulfovibrio salexigens	*Desulfovibrio*	47.0	47.7	37	Mud
Desulfovibrio vulgaris DP4	*Desulfovibrio*	63.0	77.3	37	Sediment
Desulfovibrio vulgaris Hildenborough	*Desulfovibrio*	63.1	77.2	37	Soil
Desulfovibrio vulgaris Miyazaki	*Desulfovibrio*	67.1	85.9	37	Fresh water, Salt marsh, Soil
Escherichia coli	Gammaprotebacteria	50.8	54.3	37	Host, Human intestinal microflora
Photobacterium profundum	Gammaprotebacteria	41.9 ; 41.2*	34.1 ; 35.0*	10	marine
Shewanella oneidensis	Gammaprotebacteria	45.9	44.5	30	Fresh water, Sediment
Shewanella piezotolerans	Gammaprotebacteria	43.3	38.0	15-20	Deep sea, Marine, Sediment (1914m)
Shewanella violacea	Gammaprotebacteria	44.7	43.7	8	Mud, Sediment (5110m)
Vibrio fischeri	Gammaprotebacteria	38.9 ; 37.0*	26.2 ; 26.0*	30	Fresh water
Vibrio vulnificus	Gammaprotebacteria	46.4 ; 47.2*	45.6 ; 48.9*	25	Fresh water, Host
Methanococcus aeolicus	Euryarchaeota	30.0	19.7	42	Marine, Sediment
Pyrococcus abyssi	Euryarchaeota	44.7	48.6	96	Marine, Deep sea (3500m)
Pyrococcus horikoshii	Euryarchaeota	41.8	40.8	98	Hydrothermal vent, Marine (1395m)
Thermococcus barophilus	Euryarchaeota	41.7	40.5	85	Deep sea, Hydrothermal vent, Marine (3550m)
Thermococcus sibiricus	Euryarchaeota	40.2	35.7	78	Hot spring (2350m)

Piezophilic organisms are underlined.
* Chr 1 ; Chr 2.
1 OGT stands for optimal growth temperature, data from the literature or the GOLD website
2 Habitat data from GOLD website
