# Supplementary material for: The First Genomic and Proteomic Characterization of a Deep-Sea Sulfate Reducer: Insights into the Piezophilic Lifestyle of Desulfovibrio piezophilus
Source: PLoS One. 2013 Jan 30;8(1):e55130. doi: 10.1371/journal.pone.0055130 (PMC3559428; doi:10.1371/journal.pone.0055130)
Supplement: Table S7 — List of oligonucleotide primers used. (PDF) [file pone.0055130.s010.pdf]

Table S7. List of oligonucleotide primers used

Oligonucleotide	Target locus	Sequence
DESPIv2_10200-LEFT	DESPIv2_10200 (OmpH)	AGGACAGCGTTGTTGCTTTT
DESPIv2_10200-RIGHT	DESPIv2_10200 (OmpH)	GCATACAAAACTCCCCCAGA
DESPIv2_10492-LEFT	DESPIv2_10492 (ExtraC BP)	GCGAAAGAGTATCCCGATCA
DESPIv2_10492-RIGHT	DESPIv2_10492 (ExtraC BP)	CGATTCTGCAACCATCCTTT
DESPIv2_10600-LEFT	DESPIv2_10600 (GlnH)	AACAGGCTATGATGGCGAAG
DESPIv2_10600-RIGHT	DESPIv2_10600 (GlnH)	GGCTCATCGGAGATGAAATC
DESPIv2_10610-LEFT	DESPIv2_10610 (GlnH)	AGCTGCCGGTGTACTTGTCT
DESPIv2_10610-RIGHT	DESPIv2_10610 (GlnH)	CCAACATCGGGATACGATTC
DESPIv2_10834-LEFT	DESPIv2_10834 (OmpH)	TCAGGAAGACCTCGCTCATT
DESPIv2_10834-RIGHT	DESPIv2_10834 (OmpH)	TCATATGAACGGGCAGACAA
DESPIv2_10845-LEFT	DESPIv2_10845 (ArgF)	GCTGCAACTGGAGCACATTA
DESPIv2_10845-RIGHT	DESPIv2_10845 (ArgF)	CAGTGCATGAATTTGGCATC
DESPIv2_11010-LEFT	DESPIv2_11010 (CytC)	GAGGTTGCCAGTGGAATGAT
DESPIv2_11010-RIGHT	DESPIv2_11010 (CytC)	TCCTTGGCAATACCCTTGAG
DESPIv2_11075-LEFT	DESPIv2_11075 (DegP)	GGTCAGGGTTCGTCATCACT
DESPIv2_11075-RIGHT	DESPIv2_11075 (DegP)	TGATGACTGCGAGGTCTGTC
DESPIv2_11220-LEFT	DESPIv2_11220 (specific cluster)	CTGACAATGACACGGATTGG
DESPIv2_11220-RIGHT	DESPIv2_11220 (specific cluster)	AGGATGGAGGATGAGCCTTT
DESPIv2_11412-LEFT	DESPIv2_11412 (HynA)	TGGGAAACTGTGGAGAAAGG
DESPIv2_11412-RIGHT	DESPIv2_11412 (HynA)	GATTGTGGCCAATGCTTTTT
DESPIv2_11808-LEFT	DESPIv2_11808 (QmoA)	GGCACCAAAGGAAACTACGA
DESPIv2_11808-RIGHT	DESPIv2_11808 (QmoA)	ATGGGTCTTGTAAGCCATGC
DESPIv2_11809-LEFT	DESPIv2_11809 (AprA)	AGCGTGTGTTCATCGTCAAG
DESPIv2_11809-RIGHT	DESPIv2_11809 (AprA)	ACACGTTAACAGCACCACCA
DESPIv2_11824-LEFT	DESPIv2_11824 (CydB-like)	CTTGGCAGAGCCTATTCCTG
DESPIv2_11824-RIGHT	DESPIv2_11824 (CydB-like)	ACTCCCAGGTTGATGGTCAG
DESPIv2_11838-LEFT	DESPIv2_11838 (specific cluster)	TGGGAAAGAAGGCATGAAAG
DESPIv2_11838-RIGHT	DESPIv2_11838 (specific cluster)	CAGGGGCATACTGTCTCGAT
DESPIv2_11888-LEFT	DESPIv2_11888 (CydA)	CGCTGATCTTTTCCGTCTTC
DESPIv2_11888-RIGHT	DESPIv2_11888 (CydA)	AGGACATTGCCATCTTCACC
DESPIv2_12106-LEFT	DESPIv2_12106 (Unknown)	ATGGCTGTTTACCCATCGTC
DESPIv2_12106-RIGHT	DESPIv2_12106 (Unknown)	GAACTCATCCCAGCCAAAAA
DESPIv2_12413-LEFT	DESPIv2_12413 (OmpH)	AGCAGCGTATTGTTGCACTG
DESPIv2_12413-RIGHT	DESPIv2_12413 (OmpH)	GCTTGGAGAAGTCCAGTTCG
DESPIv2_12562-LEFT	DESPIv2_12562 (HisG)	CCTCGAAGGCAAGAAAGTTG
DESPIv2_12562-RIGHT	DESPIv2_12562 (HisG)	ATTCGCACGGATAGTGGTTC
DESPIv2_20109-LEFT	DESPIv2_20109 (Unknown)	GGGCGGTATTGAGGAATTTT
DESPIv2_20109-RIGHT	DESPIv2_20109 (Unknown)	CATGGAACGGACGACTTTTT
DESPIv2_16S-LEFT	DESPIv2_16S_rRNA_2	TGGGGAGCAAACAGGATTAG
DESPIv2_16S-RIGHT	DESPIv2_16S_rRNA_2	CACATACTCCACCGCTTGTG
