# Supplementary material for: The First Genomic and Proteomic Characterization of a Deep-Sea Sulfate Reducer: Insights into the Piezophilic Lifestyle of Desulfovibrio piezophilus
Source: PLoS One. 2013 Jan 30;8(1):e55130. doi: 10.1371/journal.pone.0055130 (PMC3559428; doi:10.1371/journal.pone.0055130)
Supplement: Table S8 — The genomic islands in Desulfovibrio species. (PDF) [file pone.0055130.s011.pdf]

Table S8. The genomic islands in *Desulfovibrio* species

Organism	Begin	End	Length
D. aespoeensis	(11 GIs, 7.08% of genomes)		
GI-1	441024	477079	36056
GI-2	501798	525647	23850
GI-3	910367	923183	12817
GI-4	1640011	1651942	11932
GI-5	1757128	1850059	92932
GI-6	2072450	2087931	15482
GI-7	2136780	2151318	14539
GI-8	2595084	2612005	16922
GI-9	2729061	2743106	14046
GI-10	3022190	3031081	8892
GI-11	3474382	3484989	10608
D. alaskensis	(7 GIs, 7.83% of genomes)		
GI-1	918776	1001794	83019
GI-2	1788034	1803997	15964
GI-3	1898670	1998527	99858
GI-4	2779086	2793263	14178
GI-5	2864391	2896476	32086
GI-6	2967729	2983169	15441
GI-7	3364112	3395762	31651
D. desulfuricans	(6 GIs, 3.64% of genomes)		
GI-1	280583	291348	10766
GI-2	303980	334313	30334
GI-3	520396	532874	12479
GI-4	1478173	1497715	19543
GI-5	2685449	2707059	21611
GI-6	2828124	2837947	9824
D. magneticus	(14 GIs, 9.27% of genomes)		
GI-1	82121	124966	42846
GI-2	991191	1008448	17258
GI-3	1056473	1168426	111954
GI-4	1617597	1626830	9234
GI-5	1657572	1667191	9620
GI-6	2047966	2062440	14475
GI-7	2688976	2697750	8775
GI-8	3039660	3051424	11765
GI-9	3062917	3072549	9633
GI-10	3273590	3303527	29938
GI-11	3417826	3449537	31712
GI-12	3607954	3655044	47091
GI-13	4086559	4192296	105738
GI-14	4689779	4726722	36944
D. salexigens	(2 GIs, 0.54% of genomes)		
GI-1	327557	340706	13150
GI-2	2205128	2215107	9980
D. vulgaris DP4	(4 GIs, 3.94% of genomes)		
GI-1	101089	110797	9709

	GI-2	1047313	1095258	47946
	GI-3	1765341	1787249	21909
	GI-4	2990704	3047710	57007
D. vulgaris Hildenborough	(5 GIs, 3.64% of genomes)			
	GI-1	1404246	1413142	8896
	GI-2	1580466	1595902	15436
	GI-3	2076256	2133258	57002
	GI-4	2268224	2281869	13645
	GI-5	2799302	2834336	35034
D. vulgaris Miyazaki	(5 GIs, 2.60% of genomes)			
	GI-1	94764	121776	27013
	GI-2	869406	886434	17029
	GI-3	2022521	2055510	32990
	GI-4	2169795	2182956	13162
	GI-5	3439993	3454952	14960
