# Supplementary material for: The First Genomic and Proteomic Characterization of a Deep-Sea Sulfate Reducer: Insights into the Piezophilic Lifestyle of Desulfovibrio piezophilus
Source: PLoS One. 2013 Jan 30;8(1):e55130. doi: 10.1371/journal.pone.0055130 (PMC3559428; doi:10.1371/journal.pone.0055130)
Supplement: Text S1 — Supplemental information on Materials and Methods. (DOC) [file pone.0055130.s012.doc]

**Text S1 :** Supplemental information on Materials and Methods.

**Genomic DNA preparation and sequencing.** *Desulfovibrio piezophilus* C1TLV30^T^ was grown anaerobically at 30 °C and 0.1 MPa, for 40 h in 300 mL of the medium described by Khelaifia *et al.* [1]. Total genomic DNA was extracted from the cells using the Wizard Genomic DNA purification Kit (Promega). Genome sequencing was performed by the Genoscope (CEA, Evry, France) using 454-sequencing method (Roche GS-FLX, Titanium system) and Solexa technology. A mate paired genomic library with 8 kb insert size was prepared according to the manufacturer's protocols (454 Life Sciences Corporation, Branford, CT, USA). The obtained fragments were sequenced and around 19-fold coverage of GS FLX (Titanium, [www.roche.com](http://www.roche.com/)) reads were assembled using Newbler. Gaps between contigs were closed by using the Consed editing software (www.phrap.org/), custom primer walks, and/or PCR amplification. For the quality assessment, around 230-fold coverage of Illumina reads (36 bp) were mapped onto the whole genome sequence using SOAP ([http://soap.genomics.org.cn](https://irdmail.fr/Redirect/soap.genomics.org.cn)).

**Structural and functional genome analyses.** Functional classes were classified in COGs (Clusters of Orthologous Group of proteins) by the COGnitor software imbedded into the MicroScope system. Metabolic pathways were analysed with KEGG database [2]. Prediction of putative prophages was performed using the Prophage Finder tool (http://bioinformatics.uwp.edu/~phage/ProphageFinder.php). IS elements and IS transposases were detected by Blast comparison to the ISFinder database (<http://www-is.biotoul.fr/is.html>)with manual inspection of the surrounding significant search hits. For genomic islands, SIGI-HMM [3], IslandPath-DIMOB [4] and alien Hunter [5] methods were used for codon usage bias, dinucleotide frequency and variable length of k-mers analyses, respectively. Circular maps of the chromosome was drawn using the Circos software [6]. GC content, GC skew, tetranucleotide frequency, codon usage and amino acid composition were analyzed by self-written perl scripts. The relative tetranucleotide abundance and comparison were calculated as the Karlin's method [7]. Clustering trees based on amino acid composition was constructed with PHYLIP 3.66 package [8] using NJ method. Isoelectric point (pI) of proteins was calculated using the bioperl pI calculator module with the pK values of charged amino acids assigned by EMBOSS pKa set.

**Protein samples preparation.** Protein extraction conditions were optimized as follows in order to obtain high-resolution 2D gel electrophoresis. The cell pellets were suspended with 10 mL of ice-cold sterile water. The samples were subjected to two passages in French Press (1000 psi). Disrupted cell fractions were centrifuged at 13,000 x g for 5 min at 4 °C to remove intact cells. The supernatants were centrifuged at 35,000 x g for 1 h at 4 °C to separate the soluble cytoplasmic-periplasmic and the cellular membrane fractions. Soluble proteins obtained in the supernatant were then lyophilized and suspended in ultra pure water to a final concentration of 20 µg/µL. Proteins were quantified using the Quick Start Bradford Dye Reagent (Biorad), with BSA as the standard. For 1D gel electrophoresis of proteins from the membrane fraction, the ultra-centrifuged pellets were resuspended in water, solubilized in Laemmli buffer, boiled at 100 °C for 5 min, and then 20 μg/wells proteins were deposited and separated on 12.5% polyacrylamide gel electrophoresis [9]. The Unstained Protein Molecular Weight Marker (Fermentas Life Sciences) was used as molecular marker. Gels were subsequently revealed by silver staining (PlusOne Silver staining kit, GE Healthcare).

**2D gel electrophoresis.** The first dimension (isoelectric focusing) was run in a Multiphor II IEF system (Amersham Biosciences) at 20 °C. Immobiline DryStrips (Amersham Biosciences) pH 4–7 and 6–11 (18 cm long) were used as ready-made gel strips with immobilized pH gradients. IPG-DryStrips were hydrated overnight with 350 µL of rehydratation solution (8 M urea, 2% CHAPS (w/v), 18 mM DTT, 2% IPG buffer pH 4–7 or 0.5% IPG buffer pH 6–11) containing the proteins samples (100 µg). Running conditions were set according to the IPG-DryStrips Manufacturer’s guidelines. For the second dimension, SDS-PAGE was carried out according to Laemmli [9], using 10–18% polyacrylamide slabs gradient gels containing 0.1% SDS (w/v) in a Protean II Multicell unit (Biorad). Preliminary investigations using pH 4-7 or pH 6-11 IPG strips revealed that most of the protein spots were focused on the pH 4-7 strips. Only four protein spots were detected when using pH 6-11 IPG strips. Thus, we chose to use the pH 4-7 strips for subsequent investigations.

**DiGE analysis.** All samples were washed with the 2D-Clean-up kit (GE Healthcare) according to the manufacturer’s instruction and solubilised in lysis buffer to a final concentration of 2.5 µg/µl (8 M urea, 2M thiourea, 4% (w/v) CHAPS, pH 8.5 without DTT and carrier ampholytes to prevent reactions between the NHS esters of the cyanine dyes and the respective thiol and primary amine groups). The labeling reaction was performed following the manufacturer’s recommendation (GE Healthcare). A stock solution of 1 ng fluor dye/µl (freshly dissolved in anhydrous dimethyl formamide ultrapure; DMF SIGMA) was diluted to a working solution of 400 pmol/µl. 50 µg of protein of either sample were labeled using 400 pmol dye. As an internal standard to be loaded on each gel, a pool of the samples (containing 25 of each individual extract) was prepared in parallel and labeled with Cy2. Samples were vortexed and incubated on ice for 30 min in the dark. The reaction was stopped by adding 1 µl of 10 mM lysine per 400 pmol dye used. Samples were vortexed and further incubated for 10 more min on ice in the dark. Labeled samples were then combined according to the experimental design. The combined samples were supplemented with an equal volume of 2x sample buffer (8 M urea, 2 M thiourea, 4% (w/v) CHAPS, 1% (v/v) IPG Buffer 3-10 (GE Healthcare). This mixture was immediately used for isoelectric focusing IEF. For the first dimension, Immobiline DryStrips (GE Healthcare) pH 3–10 (13 cm long) were used as ready-made gel strips with immobilized pH gradients. They were rehydrated with the Destreak rehydratation solution (0,5% ampholyte). Sample’s were applied using the cup-loading technique. The IPGphorII system of GE Helthcare has been used (total Vh = 48,000). Equilibration step was performed according to Taylor and Pfeiffer [10]. For the second dimension, SDS-PAGE was carried out according to Laemmli using 12% polyacrylamide gels [9]. A Typhoon Imager was used to scan the gels and the DeCyder 2D differential analysis software v6.5 (GE Healthcare) was used to analyze the profiles. Cropped images were imported onto decyder database. Then a DIA analysis of gels was performed so that exclusion filter can be defined. Batch processor was then used to gels analysis, performed according to the following steps: spot detection estimated at 10,000; exclusion volume filter for spot volume < 30,000; Master gel auto selection; differential analysis (2 groups’ comparison using the T-Test between high and low hydrostatic pressure). Differential spots were selected for a ratio > 100% and a p-value < 5%. For each culture condition, experiments were conducted in triplicate: proteins were extracted from three independent cultures, and for each of them, three 2DE-gels were run and compared. Each protein spot was considered for further analysis only if it was detected on all nine 2DE- gels.

**MALDI-TOF and LC-ESI-MS/MS mass spectrometry.** For protein identification by mass spectrometry (MS), a silver staining and destaining procedure was applied as described by Gharahdaghi et al. [11]. 1D-gel bands or 2D-gel spots were put into a 96-well microplate (Greiner) for sample digestion. A robotic workstation (Freedom EVO 100, TECAN) was used to perform automated sample preparation, including multiple steps: washes, reduction and alkylation, digestion by trypsin (Sigma, proteomics grade) and extraction of tryptic peptides. The dried digested peptides were then analyzed either by MALDI-TOF mass spectrometry in a positive reflectron mode (Microflex II, Bruker Daltonics) or by ESI-MS/MS on a LCQ-DECA XP (Thermofisher) or Q-ToF (Micromass, Waters) mass spectrometers, each one mounted with a nano spray ionization source. Protein identification was performed on the Mascot web site or by using the TurboSEQUEST algorithm26 (version 27, rev. 12) using the non-redundant NCBI database restricted to *Desulfovibrio* (48 103 entries) and the *D. piezophilus* C1TLV30^T^ database (3,354 entries). Search parameters criteria were set as: two missed cleavage sites allowed, variable methionine oxidation, cysteine carbamidomethylation and no fixed modification. Protein identification was validated when at least 2 unique peptides of rank 1 (corresponding to a protein score value ≥20 in the Bioworks Browser 3.1. SR1 software,Thermo Electron Corporation). The positive matches with 2 or 3 unique peptides were manually checked and only MS/MS spectra with the following criteria were accepted : 1- at least half of the total theoretical y and b ions were present; 2- matches should correspond to the most intense peaks ; 3- a series of at least 5 consecutive y or b ions was observed.

**RNA and cDNA preparation.** For total RNA isolation, cell pellets were rinsed three times with 10 mM Tris-HCl (pH 8.0) RNAse-free buffer and finally resuspended in 200 μL of 10 mM Tris-HCl, 1 mM EDTA (pH 8.0) RNase-free buffer. Total RNA was isolated by using the High Pure RNA Isolation Kit (Roche Diagnostics) according to the manufacturer’s instructions with an extra DNase I digestion step in order to eliminate contaminating DNA. RNAs quality was checked by electrophoresis on agarose gel and the absence of DNA contamination was confirmed by PCR. RNAs was quantified to the spectrophotometer at 260 nm (NanoDrop 1000 ThermoScience). For cDNA synthesis, 3 μg of total RNAs and 3 μg of random primers (Invitrogen) were mixed, heated to 70 °C for 3 min and placed in ice. The cDNA synthesis mix [50 mM Tris-HCl (pH 8.3), 40 mM KCl, 6 mM MgCl_2_, 10 mM DTT, and 0.3 mM dNTPs] was then added. The reaction mix (30 μL) was first incubated for 5 min at 25 °C, then 300 units of Superscript II reverse transcriptase (Invitrogen) were added followed by 5 min incubation at 25 °C, then 1 h at 42 °C and finally for 15 min at 70 °C for heat inactivation. The reaction mixture volume was then adjusted to 100 μL with ultra-pure water. cDNAs were prepared from four independent cultures and were used further for quantitative Real-Time PCR.

1. Khelaifia S, et al. (2011) *Desulfovibrio piezophilus*, sp. nov., a novel piezophilic sulfate-reducing bacterium isolated from wood falls in Mediterranean Sea. *Int J Syst Evol Microbiol* 61:2706-2711*.*
2. Kanehisa M, Goto S, Kawashima S, Okuno Y, Hattori M (2004) The KEGG resource for deciphering the genome. *Nucleic Acids Res* 32:D277-280.
3. Waack S, et al. (2006) Score-based prediction of genomic islands in prokaryotic genomes using hidden markov models. *BMC Bioinformatics* 7:142.
4. Vernikos GS, Parkhill J (2006) Interpolated variable order motifs for identification of horizontally acquired dna: revisiting the salmonella pathogenicity islands. *Bioinformatics* 22:2196–2203.
5. Hsiao W, Wan I, Jones SJ, Brinkman FSL (2003) Islandpath: aiding detection of genomic islands in prokaryotes. *Bioinformatics* 19:418–420.
6. Krzywinski M, et al. (2009) Circos: an information aesthetic for comparative genomics *Genome Res* 19:1639-1645.
7. Karlin S, Campbell AM, Mrázek J (1998) Comparative DNA analysis across diverse genomes. *Annu Rev Genet* 32:185-225.
8. Felsenstein J (1981) Evolutionary trees from dna sequences: A maximum likelihood approach. *J Mol Evol* 17:368–376.
9. Laemmli UK (1970) Cleavage of structural proteins during the assembly of the head of bacteriophage T4. *Nature* 227:680-685.
10. Taylor CM, Pfeiffer SE (2003) Enhanced resolution of glycosylphosphatidylinositol-anchored and transmembrane proteins from the lipid-rich myelin membrane by two-dimensional gel electrophoresis. *Proteomics* 3:1303-1312.
11. Gharahdaghi F, Weinberg CR, Meagher DA, Imai BS, Mische SM (1999) Mass spectrometric identification of proteins from silver-stained polyacrylamide gel: a method for the removal of silver ions to enhance sensitivity. *Electrophoresis* 20:601-605.
